# Mesenchymal stem cell-derived extracellular vesicles exert Th1-mediated anti-inflammatory effects via miR-146a/NF-κB pathway: comparison with dupilumab in a mouse model of atopic dermatitis

**DOI:** 10.1186/s13287-025-04649-z

**Published:** 2025-09-25

**Authors:** Doil Park, Joo Ho Kim, Hyeock Yang, Yeeun Ji, Jaein Yoo, Jieun Kim, Oh Young Bang

**Affiliations:** 1 Department of Health Sciences and Technology, Samsung Advanced Institute for Health Sciences and Technology (SAIHST), Sungkyunkwan University, Seoul, South Korea; 2 S&E bio, Inc. Seoul, Seoul, South Korea; 3 Department of Neurology, Samsung Medical Center, Sungkyunkwan University, 81 Irwon-ro, Gangnam-gu, Seoul, 135-710 South Korea

**Keywords:** Atopic dermatitis, Extracellular vesicles, Th cells, Dupilumab, Cytokines, Mesenchymal stem cells

## Abstract

**Background:**

Atopic dermatitis (AD) is a chronic inflammatory skin disease primarily treated with corticosteroids and dupilumab, a monoclonal antibody targeting interleukin (IL)-4 and IL-13. Mesenchymal stem cell-derived extracellular vesicles (MSC-EVs) are promising alternatives owing to their anti-inflammatory properties. This study compared the therapeutic effects of dupilumab and MSC-EVs in a murine model of AD. We employed clinical, serological, and histological analyses to assess the efficacy of these treatments and investigated their mechanisms in vitro and in vivo.

**Methods:**

We generated Wharton’s jelly MSC-EVs using a 3D microwell-based platform and evaluated their effects in an AD mouse model. AD was induced by repeated application of 2,4-dinitrochlorobenzene (DNCB) and sodium dodecyl sulfate (SDS). The mice were randomly divided into four groups: healthy (normal), placebo (DNCB + SDS), EV (6 × 10^8^ particles once), and dupilumab (25 mg/kg biweekly) groups. Dupilumab and EVs were injected subcutaneously into the dorsal skin of the mice. Dermatitis scores, serum inflammatory markers, and histological analyses were performed to evaluate disease severity and changes at the tissue level. Additionally, tumor necrosis factor (TNF)-α-induced HaCaT cells were utilized for in vitro experiments to investigate the molecular mechanisms of MSC-EV therapy.

**Results:**

EVs and dupilumab improved the clinical dermatitis score, reduced epidermal thickness, and promoted restoration of the skin barrier in mice with AD. Treatments decreased T helper (Th)2 and pro-inflammatory cytokines, but EVs, unlike dupilumab, effectively suppressed Th1 and Th22 cytokines. EVs suppress Th1 activation through the AKT/NF-κB pathway via microRNA-146a.

**Conclusions:**

MSC-EVs offer a novel cell-free therapy for AD, demonstrating comparable or superior efficacy to dupilumab, with broader immunomodulatory effects and the advantage of a single-dose administration.

**Supplementary Information:**

The online version contains supplementary material available at 10.1186/s13287-025-04649-z.

## Background

Atopic dermatitis (AD) is a chronic inflammatory skin disease caused by both genetic and environmental factors [1–3]. Although not life-threatening, AD causes persistent itching, eczematous lesions, and inflammation, affecting 3–10% of adults and approximately 20% of children worldwide [4–7]. Current treatments rely on corticosteroids, which, despite their efficacy, pose risks of skin atrophy and infection [8]. Dupilumab, a monoclonal antibody targeting interleukin (IL)-4 and IL-13, has been approved for moderate to severe AD; however, its long-term effects remain uncertain, with reports of T helper (Th)1-mediated disorders, such as psoriasis [9].

Mesenchymal stem cell (MSC)-derived extracellular vesicles (EVs) offer a promising cell-free alternative, retaining the therapeutic properties of MSCs while avoiding the associated risks. However, conventional two-dimensional (2D) MSC cultures yield low quantities of EVs with functional variability, which limits their clinical application. Strategies to enhance EV production and efficacy include external stimuli (such as hypoxia, preconditioning), three-dimensional (3D) culture systems (such as micropatterned wells, bioreactors, organoids), and EV engineering (such as encapsulation, surface modification) [10, 11]. Our previous studies demonstrated that MSCs cultured in 3D as size-regulated aggregates maintained their innate phenotype better and secreted higher levels of therapeutic EVs, including microRNAs (miR) and cytokineos, than those cultured in 2D media [12]. Additionally, scalable 3D-bioprocessing improved EV yield, reduced donor/batch variability, and enhanced therapeutic outcomes in ischemic stroke and wound-healing models [13, 14].

**Fig. 1:**
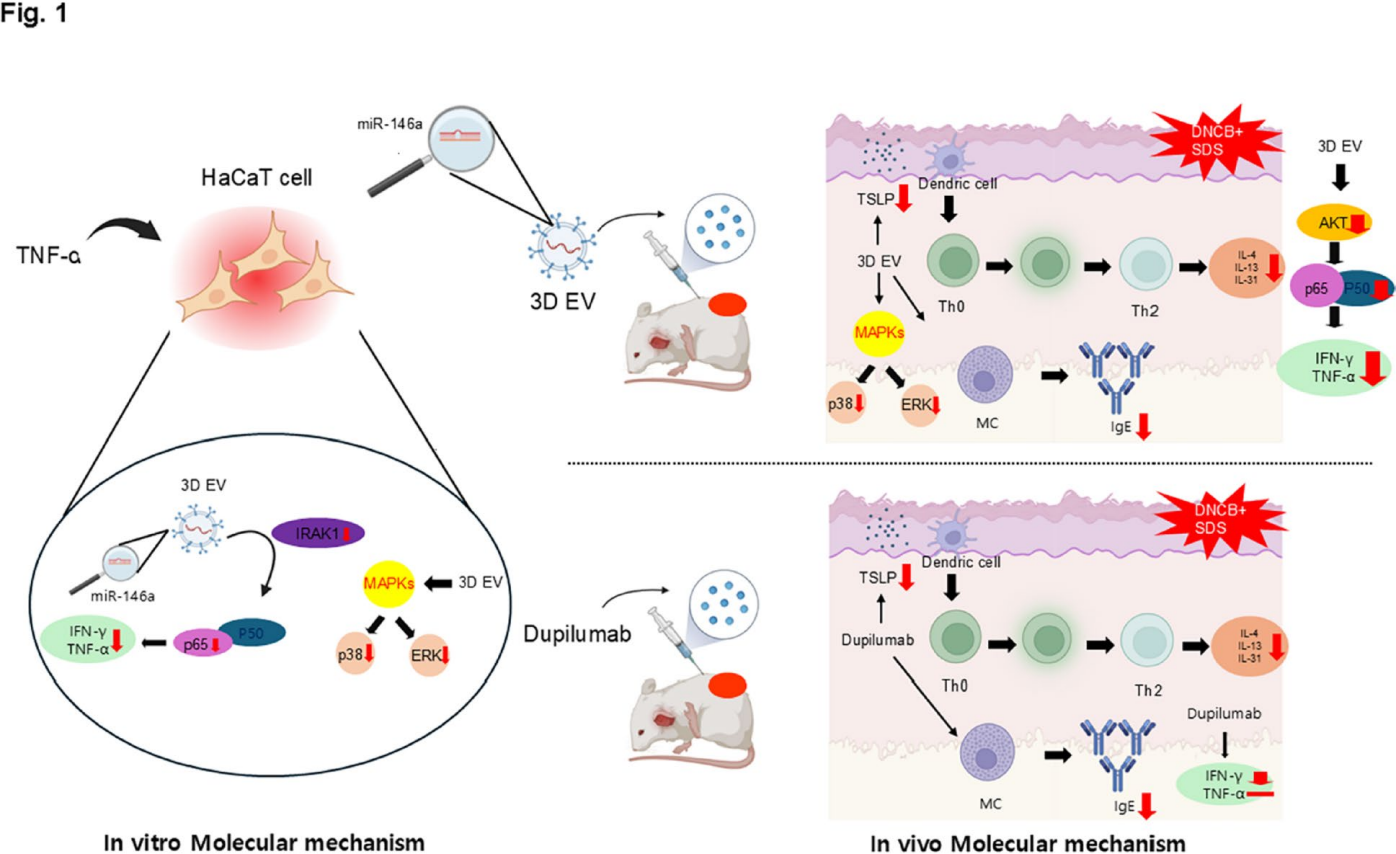
Summary of the mode of action of dupilumab and extracellular vesicles

We hypothesized that MSC-EVs and dupilumab would exert distinct therapeutic effects in an AD mouse model. To test this, we developed an EV bioprocessing platform using cell-nonadherent microwell-patterned arrays for the scalable production of Wharton’s jelly (WJ)-MSC-derived EVs in serum-free medium. We compared the effects of MSC-EVs and dupilumab through clinical, serological, and histological analyses and explored their mechanisms in both in vitro and in vivo models (Fig. 1).

## Methods

### 3D spherical culture of WJ-MSC

Human WJ-derived MSCs from the umbilical cord were cultured and expanded to passage five in a growth medium maintained in a 5% CO₂ incubator at 37 °C. At passage six, WJ-MSCs were used to generate 3D spheroid cultures. Cells were seeded into micropatterned wells, rinsed with phosphate-buffered saline (PBS), and detached enzymatically using TrypLE Express (Gibco, NY, USA). After centrifugation, a serum-free medium without heterologous proteins was added, and the cells were counted using a hemocytometer. A 60 mL cell suspension was then distributed into a microarray containing approximately 69,000 microwells, each measuring 500 μm in diameter and were 200 μm deep, coated with a 2-methacryloyloxyethyl phosphorylcholine polymer at a density of 400 cells per microwell. The 3D spheroid cultures were prepared in serum-free α-minimal essential medium without the addition of antibiotics. Spheroidal cell aggregates were formed by inducing spontaneous aggregation in a static environment and cultured for 4 days in a CO_2_ incubator at 37 °C. EV isolation was performed under sterile conditions in a biological safety cabinet using tangential flow filtration, as previously described [13, 14]. A schematic of the EV production, isolation, and quality control processes is shown in Supplementary Fig. 1.

### Characterization of EVs

According to the guidelines of the International Society for EVs (Minimal Information for Studies of EVs 2018 and 2023) and the Korean Ministry of Food and Drug Safety, EVs obtained from the WJ-MSC culture medium were thoroughly characterized in terms of structural morphology, size distribution, surface protein markers, purity, functional properties, and stability [15, 16]. Detailed methodologies for the nanoparticle tracking analysis, cryotransmission electron microscopy, transmission electron microscopy, and MACSPlex analysis are shown in Supplementary Fig. 2.

### Animal model of AD

**Fig. 2:**
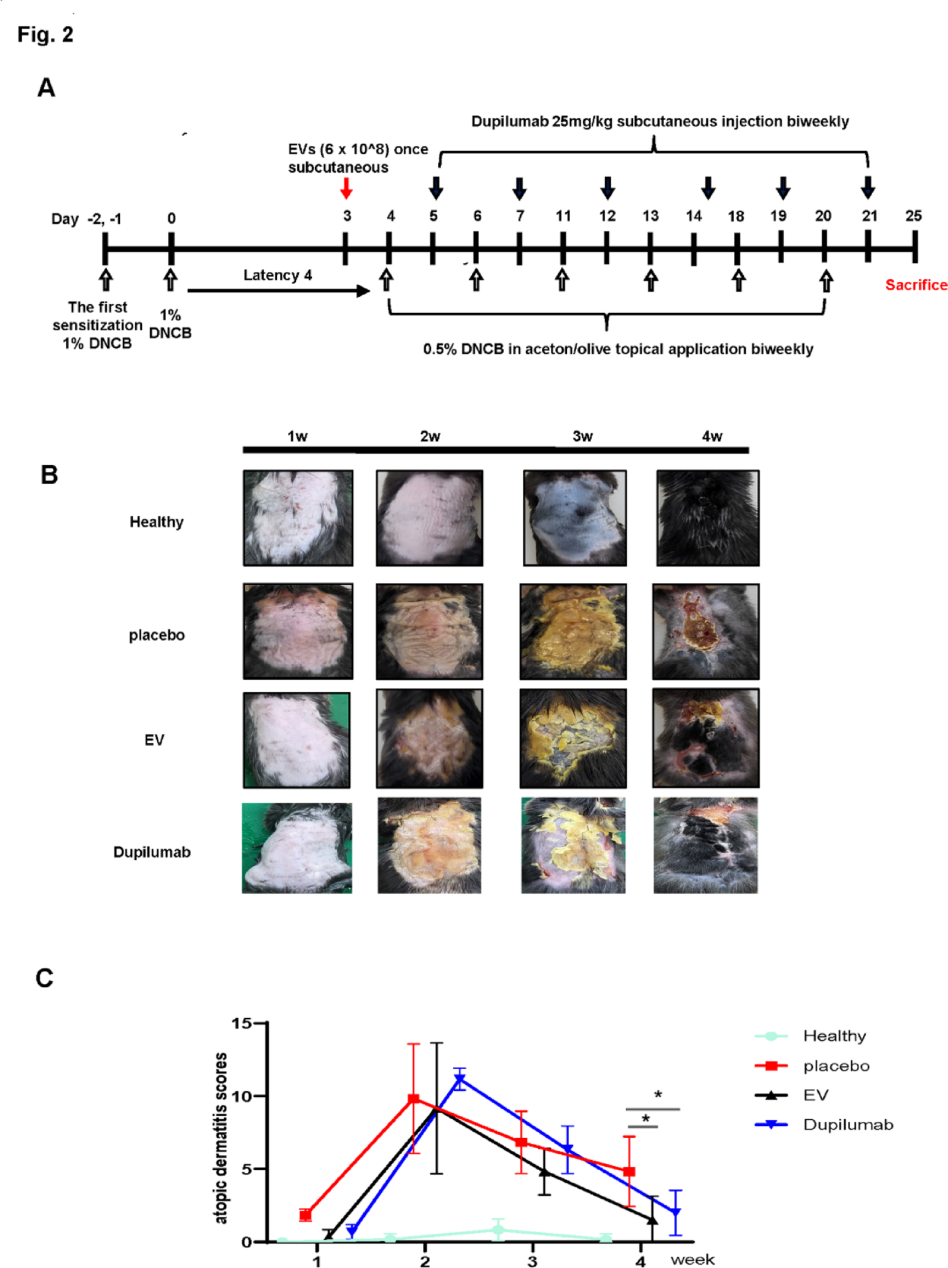
Effect of 2,4-dinitrochlorobenzene + sodium dodecyl sulphate (DNCB + SDS)-induced atopic dermatitis mice. **a** DNCB + SDS-induced atopic dermatitis mouse model treated with dupilumab and extracellular vesicles. **b** Photos of the dorsal of rats in each group for 28 days. **c** Dermatitis scores were assessed once a week from days 1 to 28

To test for skin lesions resembling AD, the shaved dorsal skin of the mice was treated with 1% 2,4-dinitrochlorobenzene (DNCB, Sigma-Aldrich Chemical Co. MO, USA; dissolved in acetone: olive oil = 3:1) once daily for the first week. After four days of rest, 0.5% DNCB solution was applied to the dorsal skin according to the experimental schedule for 21 days (Fig. 2a). A 4% sodium dodecyl sulphate (SDS) solution was used before sample treatment to enhance skin barrier disruption. The mice were randomly divided into four groups (*n* = 6 each): Healthy (normal group), placebo (DNCB + SDS), EV (EV 6 × 10^8^ particles once), and dupilumab (dupilumab 25 mg/kg, biweekly) groups. Dupilumab and EVs were subcutaneously injected into the dorsal skin of the mice. The EV dose was selected based on results from our previous mouse models of other diseases [13, 14]. The healthy control group was injected subcutaneously with PBS. The mice were sacrificed on day 25 in another experiment (Fig. 2a). After sacrifice, blood samples were collected by orbital puncture, and dorsal skin samples were collected for histological, immunohistochemical, and molecular analyses. Mice were anaesthetized with 2–3% isoflurane (Hana Pharm Co., Ltd., Seoul, Korea) delivered via a precision vaporizer during all experimental procedures. Euthanasia was performed by CO₂ inhalation (20–30% chamber volume/min fill rate) followed by cervical dislocation, in accordance with the Institutional Animal Care and Use Committee (IACUC) guidelines.

### Clinical evaluation of the severity of AD

The severity of skin lesions was assessed once a week from the first week after induction. Dermatitis scores were evaluated using a clinical visual assessment, with symptom scores ranging from 0 to 3 (0, no point; 1 point, mild; 2 points, moderate; 3 points, severe). The severity of skin lesions was reported as the total score for four symptoms: erythema, scar/dry, swelling, and erosion [17].

### Immunohistochemistry

After sacrifice, the ear and dorsal skin of each mouse were separated, fixed with 4% paraformaldehyde, and embedded in paraffin. Tissues were sectioned into 6-µm thick slices and stained with hematoxylin and eosin (H&E) to evaluate epidermal thickness and inflammatory infiltration. An H&E staining kit (ab245880) was purchased from Abcam (Cambridge, UK) and used according to the manufacturer’s instructions.

After developing an AD model, we experimented to confirm the effectiveness of EV and dupilumab treatment using the epithelial cell-derived cytokines thymic stromal lymphopoietin (TSLP) and filaggrin, a skin protein component. Back skin tissue was fixed with 4% paraformaldehyde and blocked with 10% healthy goat serum. The tissues were then incubated overnight at 4 °C with rabbit anti-TSLP (1:400; Abcam, UK) and goat anti-filaggrin (1:500; Abcam, UK) antibodies. The tissues were washed with PBS and incubated with secondary DyLight-labeled anti-chlorine Immunoglobulin (Ig)G (1:200, 594 nm; Abcam, UK) and DyLight-labeled anti-rabbit IgG (1:200, 488 nm; Vector Laboratories, Burlingame, CA, USA). Samples were imaged using a fluorescence microscope (EVOS; Advanced Microscopy Group, Bothell, WA, USA), and positively stained cells were quantified using ImageJ software.

To assess mast cell infiltration, skin sections were stained with the NovaUltraTM Toluidine Blue Stain Kit (IHCWORLD, MD, USA) according to the manufacturer’s instructions.

### Enzyme-linked immunosorbent assay (ELISA)

One gram of dorsal skin protein (*n* = 6) from pulmonary homogenates and protease inhibitor cocktails was extracted in a radioimmunoprecipitation assay (RIPA) buffer. The homogenate was centrifuged at 12,000 ×g for 30 min at 4 °C, and the supernatant was carefully removed without disturbing the pellet. The protein concentration was determined using the Bradford protein assay. The concentrations of IL-4, IL-13, IL-22, and tumor necrosis factor (TNF)-α in the supernatant were measured using an ELISA kit (BD Biosciences, New Jersey, USA), according to the manufacturer’s instructions. Optical density was measured at 450 nm using an ELISA reader (Molecular Devices, Downingtown, PA, USA).

### Western blot assay

HaCaT cells were seeded with 5 × 10^8^ EVs and incubated for 24 h. Proteins from skin homogenates and protease inhibitor cocktails were extracted using RIPA buffer. Cells were collected for each treatment concentration, and protein levels were quantified using the Bradford reagent (Bio-Rad, Hercules, CA, USA). Proteins were transferred to a nitrocellulose membrane following SDS-polyacrylamide gel electrophoresis (SDS-PAGE), then cultured in 1 × tris-buffered saline containing 0.1% Tween-20, and blocked at room temperature with skimmed milk for 1 h. Membranes were incubated overnight at 4 ℃ with primary antibodies (1:1000 dilution) for p-p65, p65, p-ERK, ERK, p-p38, p38, and β-actin (1:5000). Secondary antibodies (anti-rabbit IgG-horseradish peroxidase [HRP; IgG-HRP] and anti-mouse IgG-HRP) were then incubated at room temperature for 1 h. Protein signals were detected using the Super Signal™ West Femto Maximum Sensitivity Substrate (ThermoFisher Scientific, Lenexa, KS) and visualized using the Amersham Imager 600 (Pittsburgh GE Healthcare Life Sciences, USA).

### Cell transfection

HaCaT cells were grown to 30–60% confluence, transfected with miR-146a mimic, miR-146a inhibitor, negative control mimic (NC mimic), or negative control inhibitor (NC inhibitor) using Lipofectamine 3000 Reagent (Invitrogen, Carlsbad, CA, USA) in Opti-MEM (Invitrogen) for 6 h, and then transferred to fresh Dulbercco’s modified eagle medium containing 10% fetal bovine serum. A miR-146a mimic (cat. no. SMM-MI0000477), miR-146a inhibitor (cat. no. SMM-MI0000477), and their respective NCs were synthesized by Bioneer (Daejeon, Korea).

### Reverse transcription-quantitative polymerase chain reaction (RT-qPCR)

RNA acquisition, cDNA synthesis, and RT-PCR were performed as described previously [18, 19]. Relative mRNA expression of each target gene was normalized to that of glyceraldehyde-3-phosphate dehydrogenase (GAPDH). Total RNA was isolated from each cell line using Hybrid-RTM (GeneAll, South Korea). Total RNA (500 ng) was reverse transcribed into cDNA using oligo(dT) primers, with incubation at 55 ℃ for 60 min, followed by storage at 85 °C for 5 min and subsequently at 4 °C for further use. Real-time qPCR was performed using a Universal SYBR Green Master Mix (Applied Biosystems, USA). The cDNA was amplified using the following program conditions: 40 cycles at 95 °C for 15 min, 95 °C for 30 s, 60 °C for 30 s, and 72 °C for 30 s. Real-time PCR analysis was performed using the Applied Biosystems StepOne system (USA). The mRNA expression levels were quantified based on the relative expression of the target gene to the GAPDH gene (2-ΔCt). The primer sequences used for RT-PCR are listed in Supplementary Table 1.

### Luciferase reporter assay

The firefly luciferase reporter plasmid containing the 3ʹ-untranslated region (UTR) of interleukin 1 receptor associated kinase 1 (IRAK1) gene, along with the empty luciferase vector required for luciferase analysis was obtained from Promega. HaCaT cells were co-transfected with 50 nM miR-146a mimic or scrambled miRNA using Lipofectamine 3000 (Invitrogen). We inserted the 3ʹ UTR of IRAK1 with miR-146a binding site into the pmirGOL vector. The luminescence level was measured using the Dual-Luciferase^®^ Reporter Assay System (Promega, Madison, WI, USA). Wildtype IRAK1 3ʹ UTR: sense primer, 5ʹ-CCCCCAAATCCGGAAGTCAAAGTTCTCAG-3ʹ and antisense primer: 5ʹ-AGTATAGTGGGGGTTTAGGCCTTCAGTTTCAAGAGTCAGCT-3ʹ IRAK1 Mut primer sense: 5ʹ-CCCCCAAATCCGGAAGTCAATCAAGACAG-3ʹ, IRAK1 Mut primer antisense: 5ʹ-GGGGGTTTAGGCCTTCAGTTAGTTCTG-3ʹ. The Dual-Luciferase Reporter Assay System was used to analyze luciferase activity 48 h after transfection, following the manufacturer’s instructions.

### Statistical analysis

Statistical analysis of the data was performed using GraphPad Prism 8 software package (GraphPad Software, San Diego, CA, USA). We thank the reviewer for this important comment. Normality of the data was assessed using the Shapiro–Wilk test. Since all groups passed the normality test and displayed comparable variance,

we performed one-way ANOVA followed by Tukey’s HSD post hoc test.

## Results

### Both dupilumab and EVs reduce dermatitis symptoms in an AD model

First, we used an AD mouse model to determine the efficacy of EVs in vivo. AD was induced in C57BL/6 mice by applying DNCB and SDS to the dorsal skin twice a week, followed by twice-weekly applications to maintain the condition (Fig. 2a). Dupilumab and EV groups exhibited fewer symptoms than the placebo group, including itching, edema, excoriation, erosion, scaling, and dryness (Figs. 2b and c). Dermatitis scores were measured serially until day 25. Before treatment, no significant differences were observed between the groups; however, the scores were lower in the EV and dupilumab groups compared to the placebo group (*P* < 0.05).

### Both dupilumab and EVs improve dermatitis histologically in an AD model

**Fig. 3:**
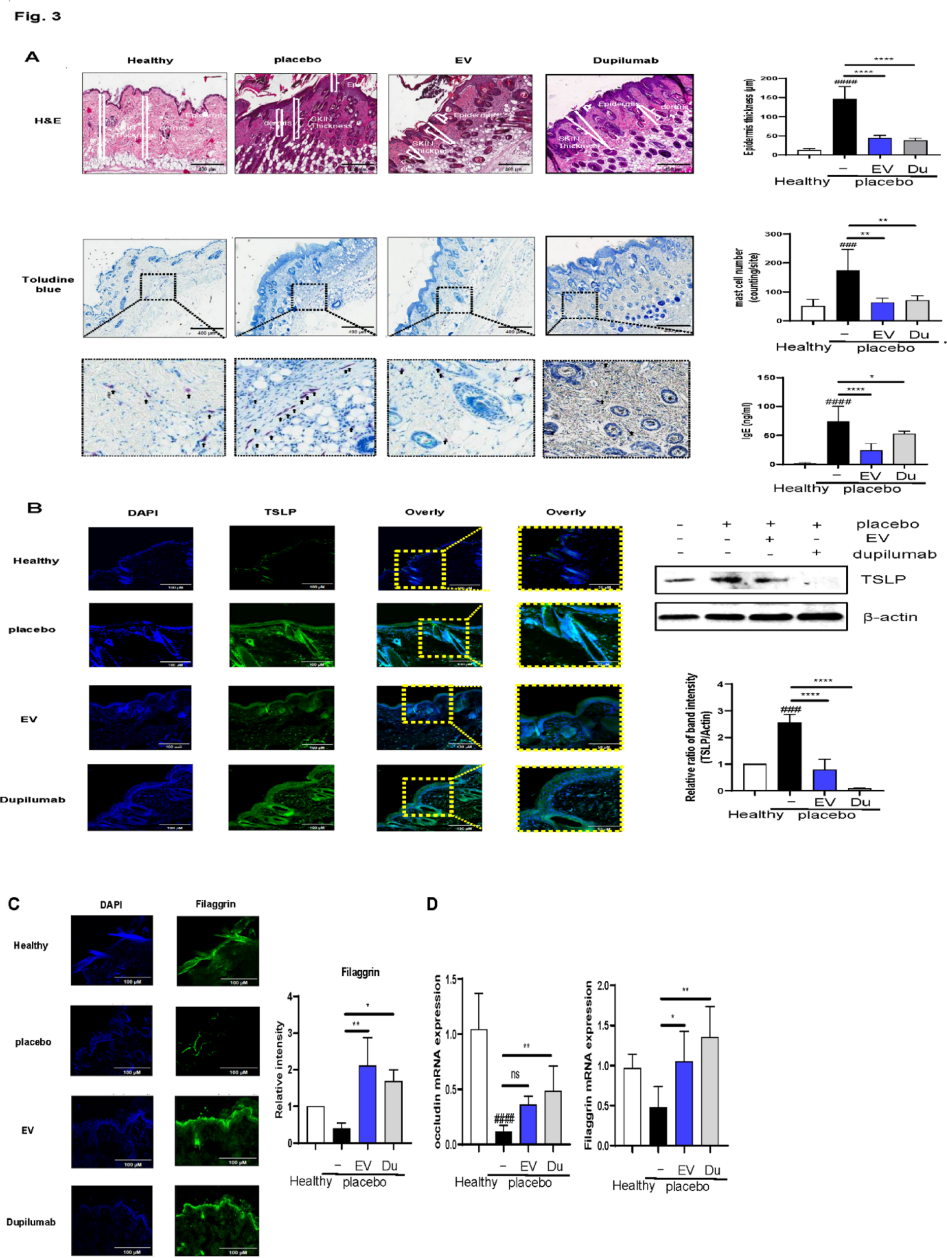
Effects of extracellular vesicles (EVs) and dupilumab on histological changes in atopic dermatitis (AD). **a** Representative histological changes of skin obtained from mice of different groups, stained with hematoxylin & eosin and toluidine blue. ELISA was used to detect the production of IgE. **b** Tissue sections were prepared and subjected to immunofluorescence staining with an anti- thymic stromal lymphopoietin (TSLP) antibody. The nuclei were counterstained with 4’,6-diamidino-2-phenylindole (DAPI, shown in blue). The effect of EVs on TSLP was assessed using western blot assay in an AD model. **c** Analysis of filaggrin (FLG) expression in the epidermal skin barrier using immunofluorescence (shown in green). Nuclear staining was performed using DAPI (blue). **d** The expression of genes in the epidermal skin barrier was estimated using real-time polymerase chain reaction analysis

We evaluated the histological effects of EV therapy. The AD model exhibited a weakened epidermal barrier, increased mast cell activation, elevated IgE levels, and a thickened epidermis. The epidermal thickness was significantly lower in the dupilumab, and EV groups compared to the placebo group (*P* < 0.0001 in both cases) (Fig. 3a). Toluidine blue staining confirmed reduced mast cell activity in both treatment groups (*P* = 0.0042 and *P* = 0.0023, respectively), with no significant difference between them. IgE levels were significantly lower in the EV group than in the placebo and dupilumab groups (*P* = 0.0266 and *P* < 0.0001, respectively) (Fig. 3a).

TSLP expression was elevated in the AD model but significantly reduced in the EV group compared to the placebo group (*P* < 0.0001) (Fig. 3b). To assess skin barrier recovery, we analyzed the expression of filaggrin and occludin. RT-qPCR confirmed their restoration in the EV and dupilumab groups compared with the placebo group (*P* = 0.0047 and *P* = 0.023, respectively) (Fig. 3c and d).

### EVs and dupilumab differentially affect T cell subsets in an AD model

**Fig. 4:**
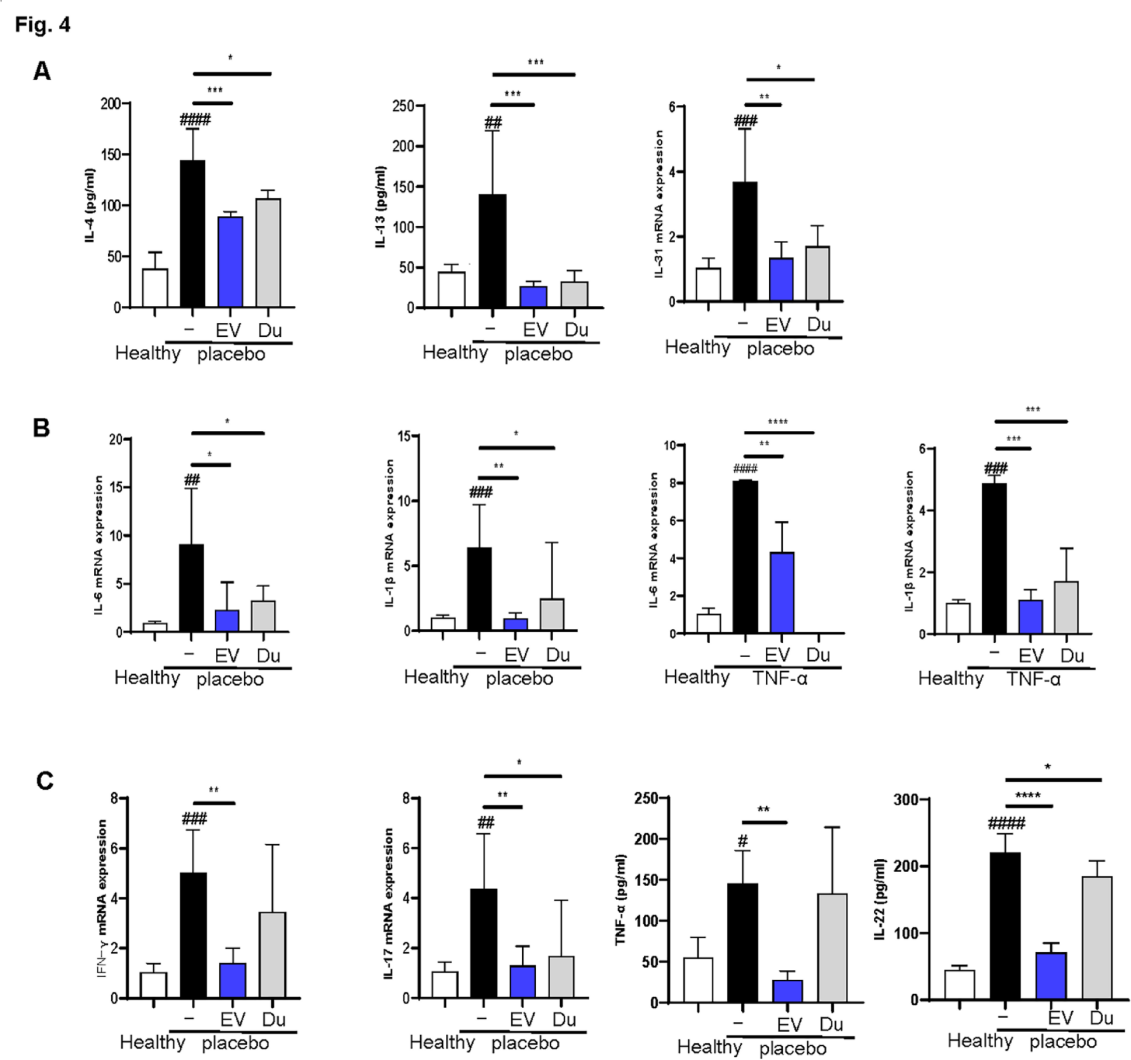
Extracellular vesicles (EVs) and dupilumab effects on mRNA expression of pro-inflammatory and T helper (Th) cytokine levels in atopic dermatitis (AD) mouse model and tumor necrosis factor (TNF)-α induced HaCaT cells. **a** Th2 cytokines were estimated using real-time polymerase chain reaction (PCR) analysis and enzyme-linked immunosorbent assay (ELISA). **b** Pro-inflammatory cytokine levels were estimated using real-time PCR analysis. **c** Effects of EVs and dupilumab on the mRNA expression of Th1, Th17, and Th22 cytokines in the dorsal skin of the AD mouse model were measured by using ELISA and real-time PCR analysis. Error bars represent the mean ± standard deviation (SD), *n* = 6

We first examined Th2-related cytokines (IL-4, IL-13, IL-31) and pro-inflammatory cytokines (IL-6, IL-1β) in an AD mouse model. ELISA and RT-qPCR showed that Th2 cytokine levels were significantly reduced in the EV group compared with those in the placebo group (*P* < 0.01 in all cases), with a similar effect in the dupilumab group (*P* < 0.05) (Fig. 4a). Pro-inflammatory cytokine levels, including IL-6 and IL-1β, were also lower in both treatment groups (*P* < 0.05 in all cases) (Fig. 4b).

Additionally, the levels of Th1, Th17, and Th22 cytokines were analyzed (Fig. 4c). While both treatments reduced these cytokines, EVs were more effective in lowering all Th1 (TNFα and IFN-γ), Th17 (IL-17) and Th22 (IL-22) cytokine levels involved in the inflammatory responses associated with AD. In contrast, dupilumab decreased the level of Th17 cytokine level (IL-17) (*P* < 0.05, in all the cases).

### EVs inhibit the protein kinase B (AKT)/nuclear factor-kappa B (NF-κB) and mitogen-activated protein kinases (MAPKs) pathways in vivo and in vitro

**Fig. 5:**
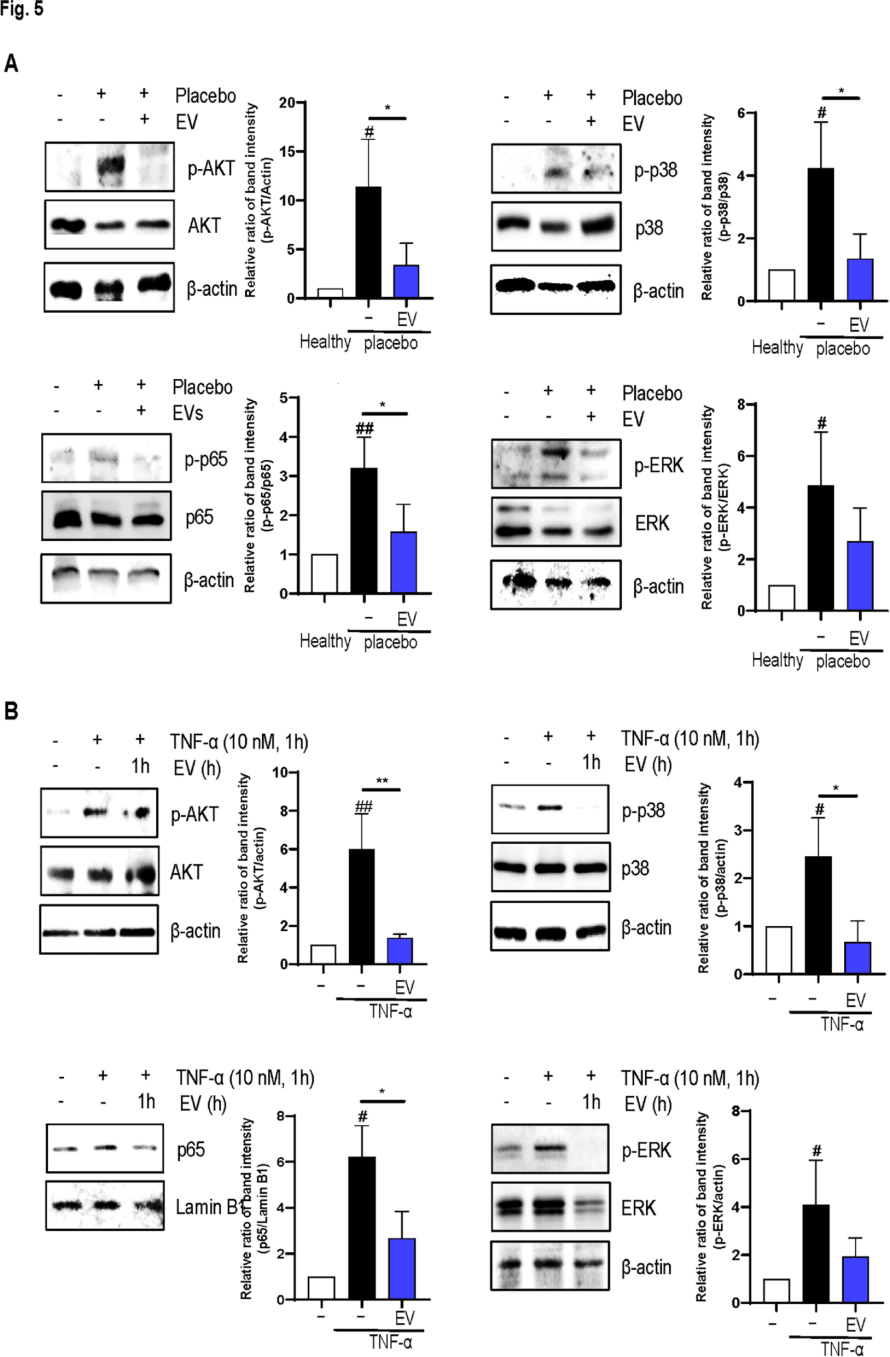
Effects of extracellular vesicles (EVs) on inflammatory pathways. Effect of EVs on AKT-NF-κB and MAPKs pathway was assessed using western blot in **a** the atopic dermatitis mouse model and **b** tumor necrosis factor-α induced HaCaT cells. Error bars represent the mean ± standard deviation (SD), *n* = 3

Western blot analysis revealed that EVs suppressed the AKT/NF-κB pathway and MAPKs pathways, a key inflammatory pathway in AD, in vivo models (*P* < 0.05 in both cases) (Fig. 5a). EVs also inhibited the AKT/NF-κB and MAPKs pathway in an in vitro model (*P* < 0.05 in all cases) (Fig. 5b).

### EVs suppress inflammation via miR-146a/NF-κB pathway

**Fig. 6:**
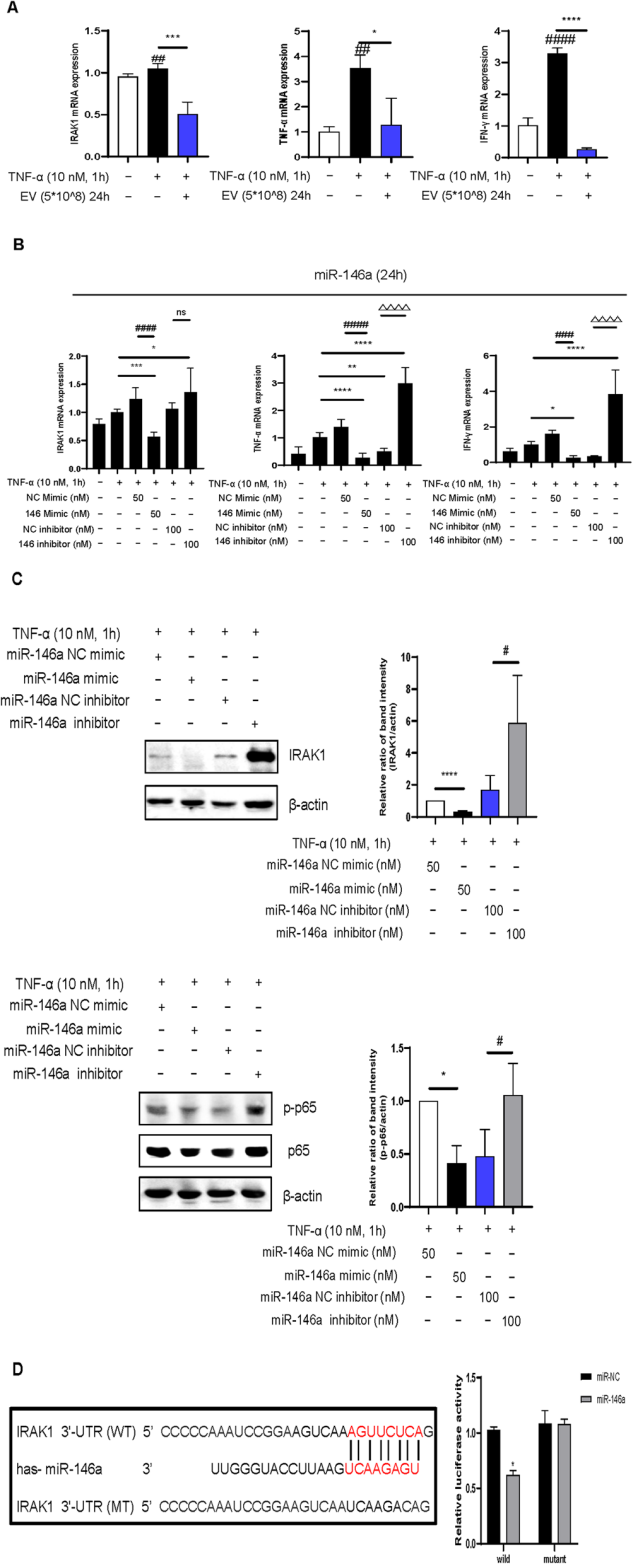
IRAK1 is the target of miR-146a. **a** Effects of extracellular vesicles on the mRNA expression of IRAK1, tumor necrosis factor (TNF)-α and interferon (IFN)-γ production in TNF-α induced HaCaT cells. **b** Reverse transcriptase-quantitative polymerase chain reaction (RT-qPCR) analysis of IRAK1, TNF-α, IFN-γ, and mRNA expression in HaCaT cells following transfection with either a miR-146a mimic or a miR-146a-3p inhibitor. **c** Western blot analysis of IRAK1 mRNA expression in HaCaT cells following transfection with either a miR-146a mimic or a miR-146a inhibitor. **d** Left panel: IRAK1 3ʹ-UTR of firefly/renilla luciferase used for microRNA reporter assay. Right panel: Quantitative data for dual-luciferase reporter assay results

To explore this mechanism, we first confirmed that EVs downregulated IRAK1, a known inflammatory target. EVs also reduced the levels of Th1 cytokines (TNF-α and IFN-γ) (Fig. 6a). miRNA profiling identified miR-146a as a key regulator, given its enrichment in EVs and its role in modulating inflammation. Transfecting HaCaT cells with a miR-146a mimic decreased IRAK1, TNF-α, and IFN-γ, whereas its inhibitor had the opposite effect (Fig. 6b and c).

Luciferase assays showed that miR-146a directly binds to the 3ʹ-UTR of IRAK1, suppressing its expression (Fig. 6d). These findings confirm that EVs regulate inflammation in AD via the miR-146a/IRAK1/NF-κB pathway.

## Discussion

This study demonstrated that clinical-scale EV therapeutics produced using a micropatterned-well system are as effective as dupilumab in reducing the clinical dermatitis score and histopathological injury in an AD mouse model. MSC-EVs are as effective as dupilumab in reducing AD severity in both in vivo (the DNCB + SDS-induced mice model) and in vitro models (TNF-α-induced HaCaT cells). Unlike dupilumab, EV therapy modulates multiple T-cell subsets (Th1, Th2, and Th17) and significantly suppresses TSLP expression. Mechanistically, EVs exert their effects via the miR-146a/NF-κB pathway. While previous studies have examined MSC-EVs in AD models [20–22], none have directly compared their effects with those of steroids or IL-4/IL-13 blockers such as dupilumab.

We investigated the mechanism of action of MSC-EVs using in vivo and in vitro models. Although the pathogenesis of AD has been attributed mainly to Th2 cells, other immune cells, including Th1, Th17, B cells, and macrophages, are also involved [23]. Inhibition of the Th2 pathway by dupilumab may lead to compensatory Th1 activation, contributing to Th1-mediated conditions, such as psoriasis. Some patients with AD initially respond well to dupilumab but later develop plaque psoriasis-like rashes, potentially due to an exaggerated response to *Demodex* or unmasked underlying dermatosis. Antagonism of the Th2 pathway by dupilumab may lead to activation of the opposing Th1 pathway in these patients, potentially resulting in Th1-mediated diseases. Initially, significant improvement and complete clearance of skin lesions were achieved with dupilumab. However, several cases have been reported of patients with AD who developed plaque psoriasis-like rashes [24–27]. The emergence of new topical dermatoses suggests the unmasking of alternative primary dermatoses in an environment where Th1 and/or Th17 activation is unresponsive. This can lead to injection-site reactions [28], possibly through an augmented reaction with *Demodex* [29, 30].

To address these limitations, we explored the broad immunomodulatory effects of EV therapy beyond Th2 inhibition. Unlike dupilumab, which explicitly targets IL-4 and IL-13, EVs regulate multiple inflammatory pathways, including the AKT-NF-κB and MAPKs pathways, which are critical for immune regulation and cell survival. miRNAs, particularly miR-146a, play a key role in the therapeutic effects of MSC-EVs [31, 32]. miR-146a, widely recognized for its role in inflammation [33–35], is induced by TLR agonists, viruses, and bacteria [36–38]. It is also upregulated in the skin of patients with psoriasis [39], indicating its involvement in inflammatory skin responses. miR-146a functions in a feedback loop by targeting key NF-κB regulators, including IRAK1, TRAF6, RELB, and CARD10 [36, 40, 41]. Our findings confirm that EV treatment reduces IRAK1 levels in keratinocytes, suppressing NF-κB signaling.

In addition to immune regulation, EV therapy also restores skin barrier integrity. Previous studies have shown that clinical-scale EV treatments enhance endothelial cell proliferation, migration, angiogenesis, and re-epithelialization while modulating inflammatory cells in wound models [14]. In the present study, we also confirmed that EVs significantly suppressed TSLP expression, which plays a crucial role in AD pathogenesis by activating dendritic and mast cells, expanding Th2 and Th22 populations and stimulating itch sensory neurons independent of Th2 cytokine levels [42–44]. TSLP is a crucial mediator of AD pathogenesis [45] and a potential drug target [46]. The expression of TSLP was significantly lower in the EV group than in the dupilumab group. Although the sample size was limited, consistent trends were observed across biological replicates, and appropriate statistical tests were used to support the significance of our findings.

## Conclusion

Our findings demonstrate that MSC-EVs offer a novel, cell-free therapy for AD, exhibiting comparable or superior efficacy to dupilumab, with broader immunomodulatory effects and the advantage of a single-dose administration. Further studies are needed to (a) compare EVs and dupilumab across different AD stages, (b) assess the potential long-term adverse effects of EVs (coagulopathy related to repetitive administration) and dupilumab (e.g., Th1-mediated disorders) [47, 48], , and (c) determine the optimal EV dosage and dose-response relationship in AD treatment. Notably, the coagulation risk may be considerably lower with local administration of EVs compared to systemic administration, and mainly when EVs are obtained during MSC spheroid cultivation, as in our study.

## Supplementary Information

Below is the link to the electronic supplementary material.

Supplementary Material 1

Supplementary Material 2

## Data Availability

The datasets generated and/or analyzed during the current study are available from the corresponding author upon reasonable request.
