# Supplementary material for: Mesenchymal stem cell-derived extracellular vesicles exert Th1-mediated anti-inflammatory effects via miR-146a/NF-κB pathway: comparison with dupilumab in a mouse model of atopic dermatitis
Source: Stem Cell Res Ther. 2025 Sep 25;16:496. doi: 10.1186/s13287-025-04649-z (PMC12465636; doi:10.1186/s13287-025-04649-z)
Supplement: Supplementary file 1 — Supplementary Material 1 [file 13287_2025_4649_MOESM1_ESM.pptx]

## Slide 1
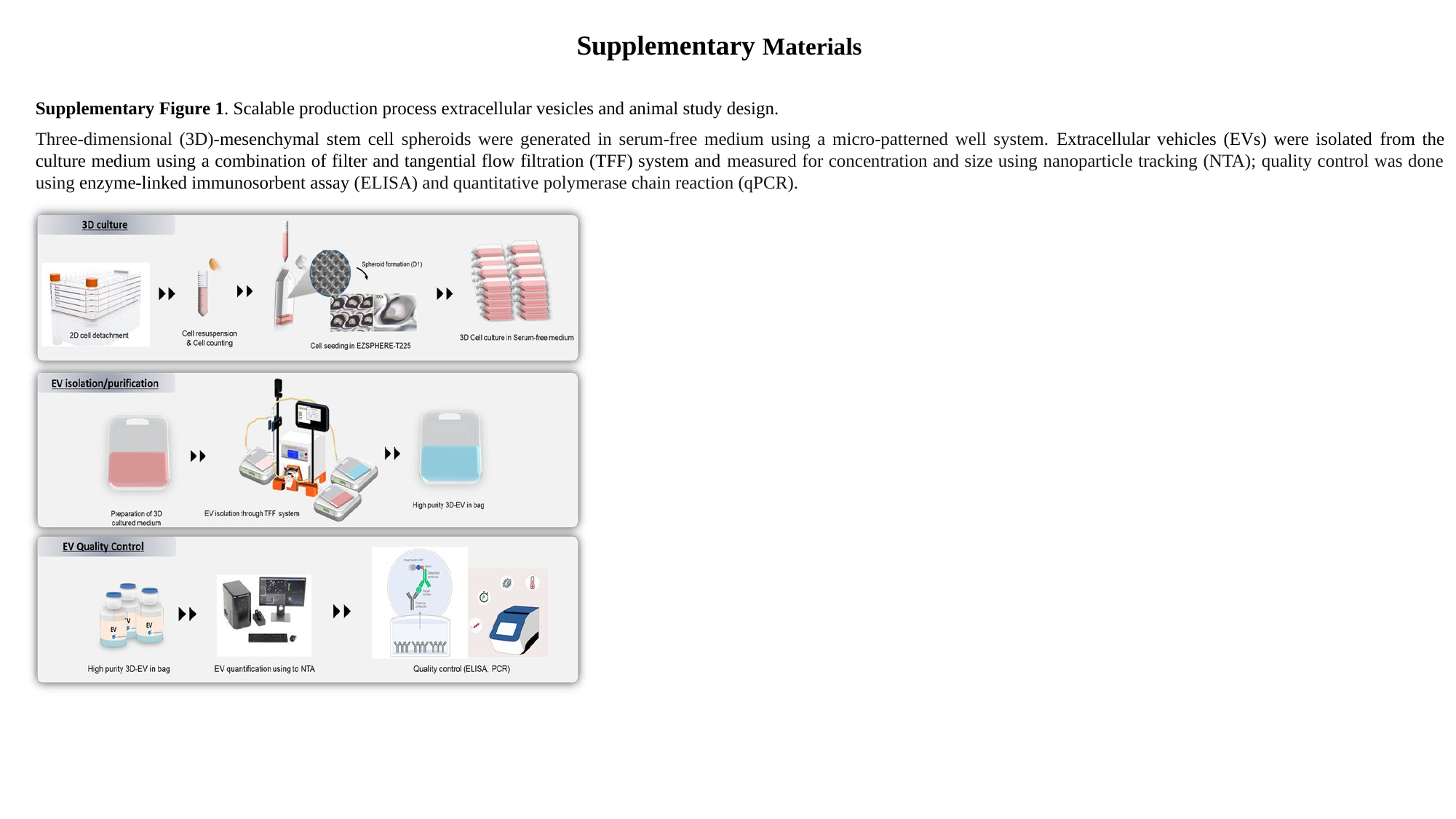

Supplementary Materials
Supplementary Figure 1. Scalable production process extracellular vesicles and animal study design.
Three-dimensional (3D)-mesenchymal stem cell spheroids were generated in serum-free medium using a micro-patterned well system. Extracellular vehicles (EVs) were isolated from the culture medium using a combination of filter and tangential flow filtration (TFF) system and measured for concentration and size using nanoparticle tracking (NTA); quality control was done using enzyme-linked immunosorbent assay (ELISA) and quantitative polymerase chain reaction (qPCR).

## Slide 2
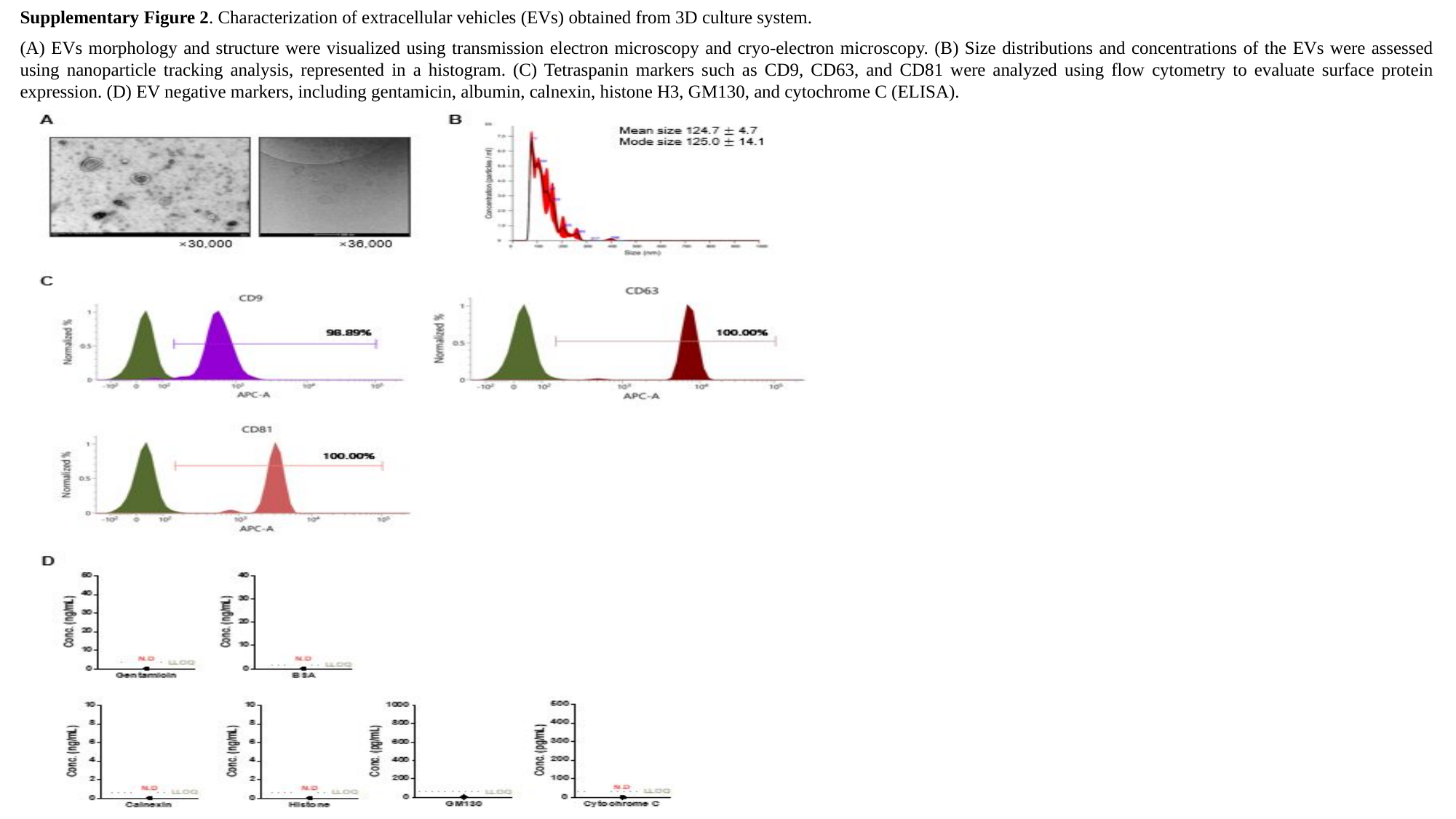

Supplementary Figure 2. Characterization of extracellular vehicles (EVs) obtained from 3D culture system.
(A) EVs morphology and structure were visualized using transmission electron microscopy and cryo-electron microscopy. (B) Size distributions and concentrations of the EVs were assessed using nanoparticle tracking analysis, represented in a histogram. (C) Tetraspanin markers such as CD9, CD63, and CD81 were analyzed using flow cytometry to evaluate surface protein expression. (D) EV negative markers, including gentamicin, albumin, calnexin, histone H3, GM130, and cytochrome C (ELISA).

## Slide 3
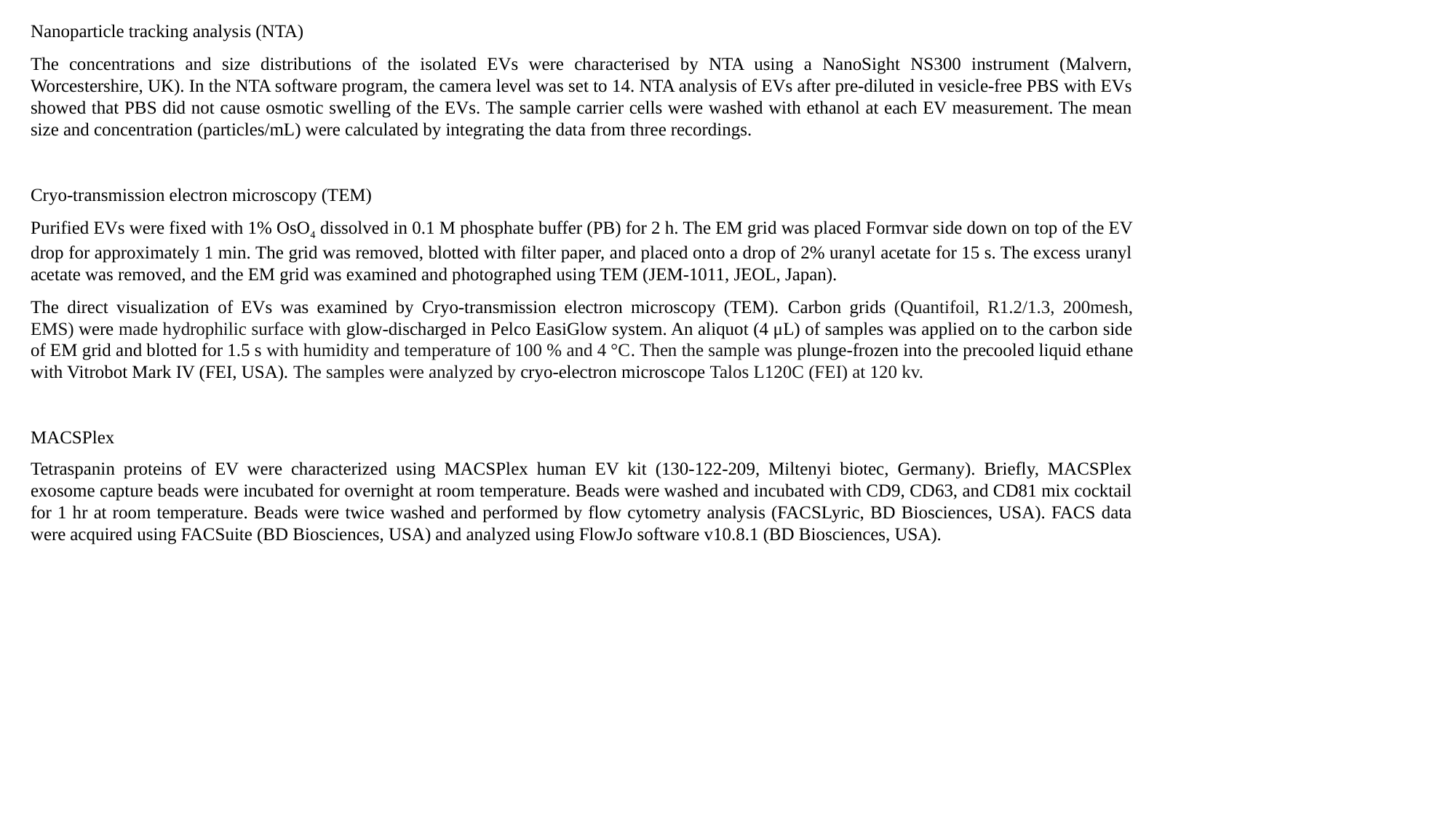

Nanoparticle tracking analysis (NTA)
The concentrations and size distributions of the isolated EVs were characterised by NTA using a NanoSight NS300 instrument (Malvern, Worcestershire, UK). In the NTA software program, the camera level was set to 14. NTA analysis of EVs after pre-diluted in vesicle-free PBS with EVs showed that PBS did not cause osmotic swelling of the EVs. The sample carrier cells were washed with ethanol at each EV measurement. The mean size and concentration (particles/mL) were calculated by integrating the data from three recordings.
Cryo-transmission electron microscopy (TEM)
Purified EVs were fixed with 1% OsO4 dissolved in 0.1 M phosphate buffer (PB) for 2 h. The EM grid was placed Formvar side down on top of the EV drop for approximately 1 min. The grid was removed, blotted with filter paper, and placed onto a drop of 2% uranyl acetate for 15 s. The excess uranyl acetate was removed, and the EM grid was examined and photographed using TEM (JEM-1011, JEOL, Japan).
The direct visualization of EVs was examined by Cryo-transmission electron microscopy (TEM). Carbon grids (Quantifoil, R1.2/1.3, 200mesh, EMS) were made hydrophilic surface with glow-discharged in Pelco EasiGlow system. An aliquot (4 μL) of samples was applied on to the carbon side of EM grid and blotted for 1.5 s with humidity and temperature of 100 % and 4 °C. Then the sample was plunge-frozen into the precooled liquid ethane with Vitrobot Mark IV (FEI, USA). The samples were analyzed by cryo-electron microscope Talos L120C (FEI) at 120 kv.
MACSPlex
Tetraspanin proteins of EV were characterized using MACSPlex human EV kit (130-122-209, Miltenyi biotec, Germany). Briefly, MACSPlex exosome capture beads were incubated for overnight at room temperature. Beads were washed and incubated with CD9, CD63, and CD81 mix cocktail for 1 hr at room temperature. Beads were twice washed and performed by flow cytometry analysis (FACSLyric, BD Biosciences, USA). FACS data were acquired using FACSuite (BD Biosciences, USA) and analyzed using FlowJo software v10.8.1 (BD Biosciences, USA).

## Slide 4
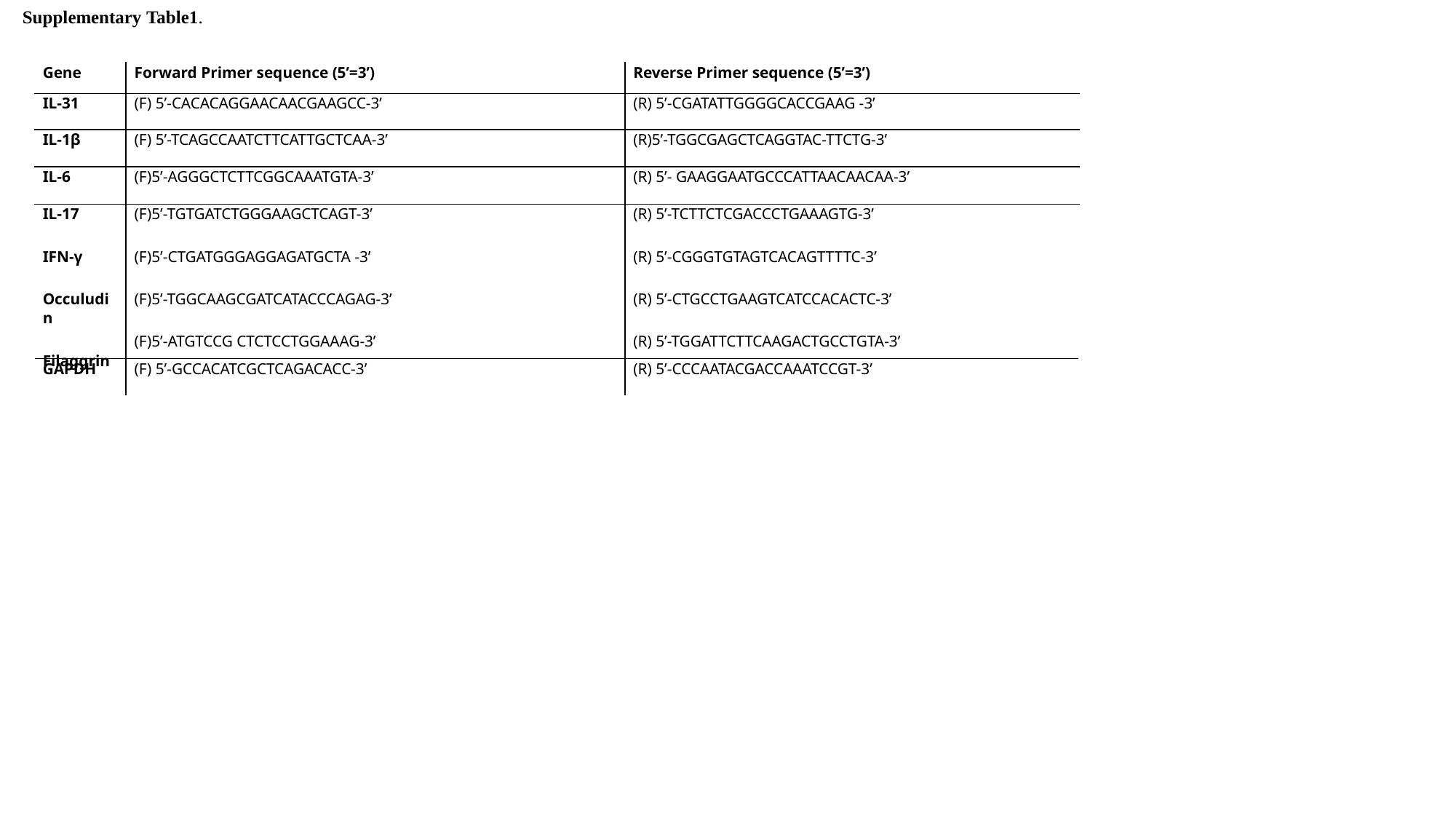

Supplementary Table1.
| Gene | Forward Primer sequence (5’=3’) | Reverse Primer sequence (5’=3’) |
| --- | --- | --- |
| IL-31 | (F) 5’-CACACAGGAACAACGAAGCC-3’ | (R) 5’-CGATATTGGGGCACCGAAG -3’ |
| IL-1β | (F) 5’-TCAGCCAATCTTCATTGCTCAA-3’ | (R)5’-TGGCGAGCTCAGGTAC-TTCTG-3’ |
| IL-6 | (F)5’-AGGGCTCTTCGGCAAATGTA-3’ | (R) 5’- GAAGGAATGCCCATTAACAACAA-3’ |
| IL-17 IFN-γ Occuludin Filaggrin | (F)5’-TGTGATCTGGGAAGCTCAGT-3’ (F)5’-CTGATGGGAGGAGATGCTA -3’ (F)5’-TGGCAAGCGATCATACCCAGAG-3’ (F)5’-ATGTCCG CTCTCCTGGAAAG-3’ | (R) 5’-TCTTCTCGACCCTGAAAGTG-3’ (R) 5’-CGGGTGTAGTCACAGTTTTC-3’ (R) 5’-CTGCCTGAAGTCATCCACACTC-3’ (R) 5’-TGGATTCTTCAAGACTGCCTGTA-3’ |
| GAPDH | (F) 5’-GCCACATCGCTCAGACACC-3’ | (R) 5’-CCCAATACGACCAAATCCGT-3’ |

## Slide 5
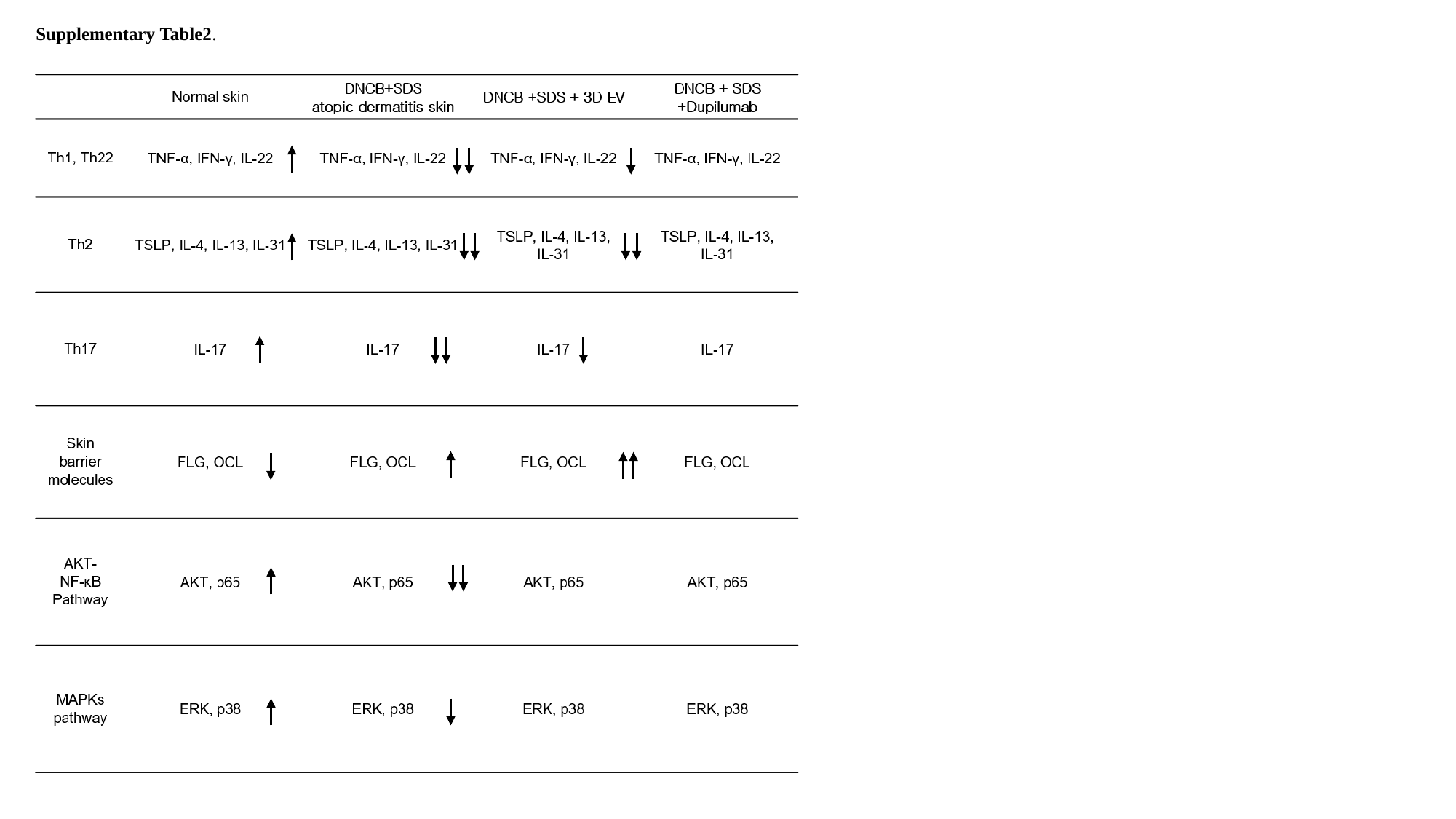

Supplementary Table2.

## Slide 6
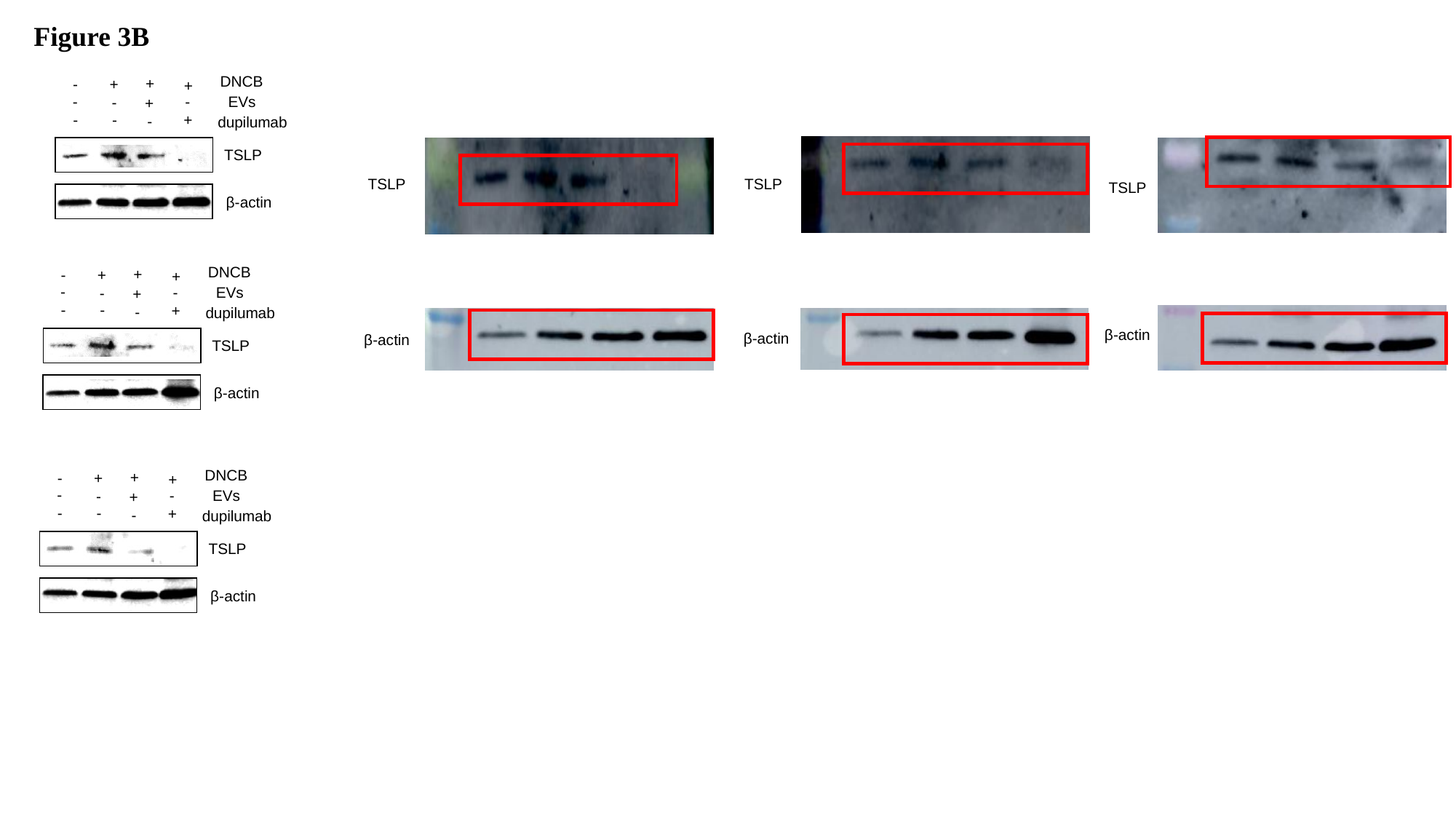

Figure 3B
DNCB
+
-
+
+
-
-
EVs
-
+
-
-
+
-
dupilumab
TSLP
β-actin
TSLP
TSLP
TSLP
DNCB
+
-
+
+
-
-
EVs
-
+
-
-
+
-
dupilumab
TSLP
β-actin
β-actin
β-actin
β-actin
DNCB
+
-
+
+
-
-
EVs
-
+
-
-
+
-
dupilumab
TSLP
β-actin

## Slide 7
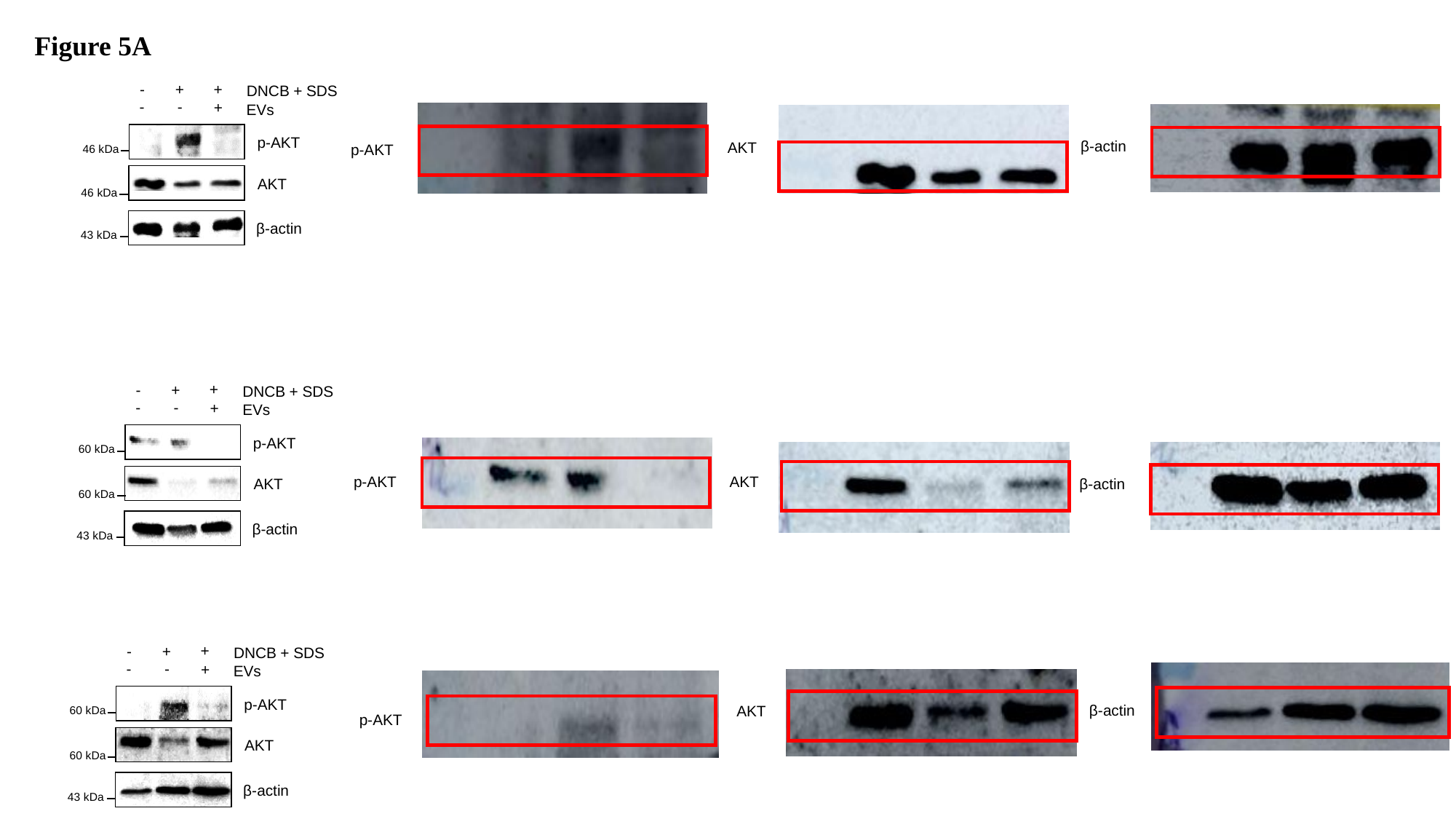

Figure 5A
+
-
+
DNCB + SDS
-
-
+
EVs
p-AKT
46 kDa
AKT
46 kDa
β-actin
43 kDa
β-actin
AKT
p-AKT
+
-
+
DNCB + SDS
-
-
+
EVs
p-AKT
60 kDa
AKT
60 kDa
β-actin
43 kDa
AKT
p-AKT
β-actin
+
-
+
DNCB + SDS
-
-
+
EVs
p-AKT
60 kDa
AKT
60 kDa
β-actin
43 kDa
β-actin
AKT
p-AKT

## Slide 8
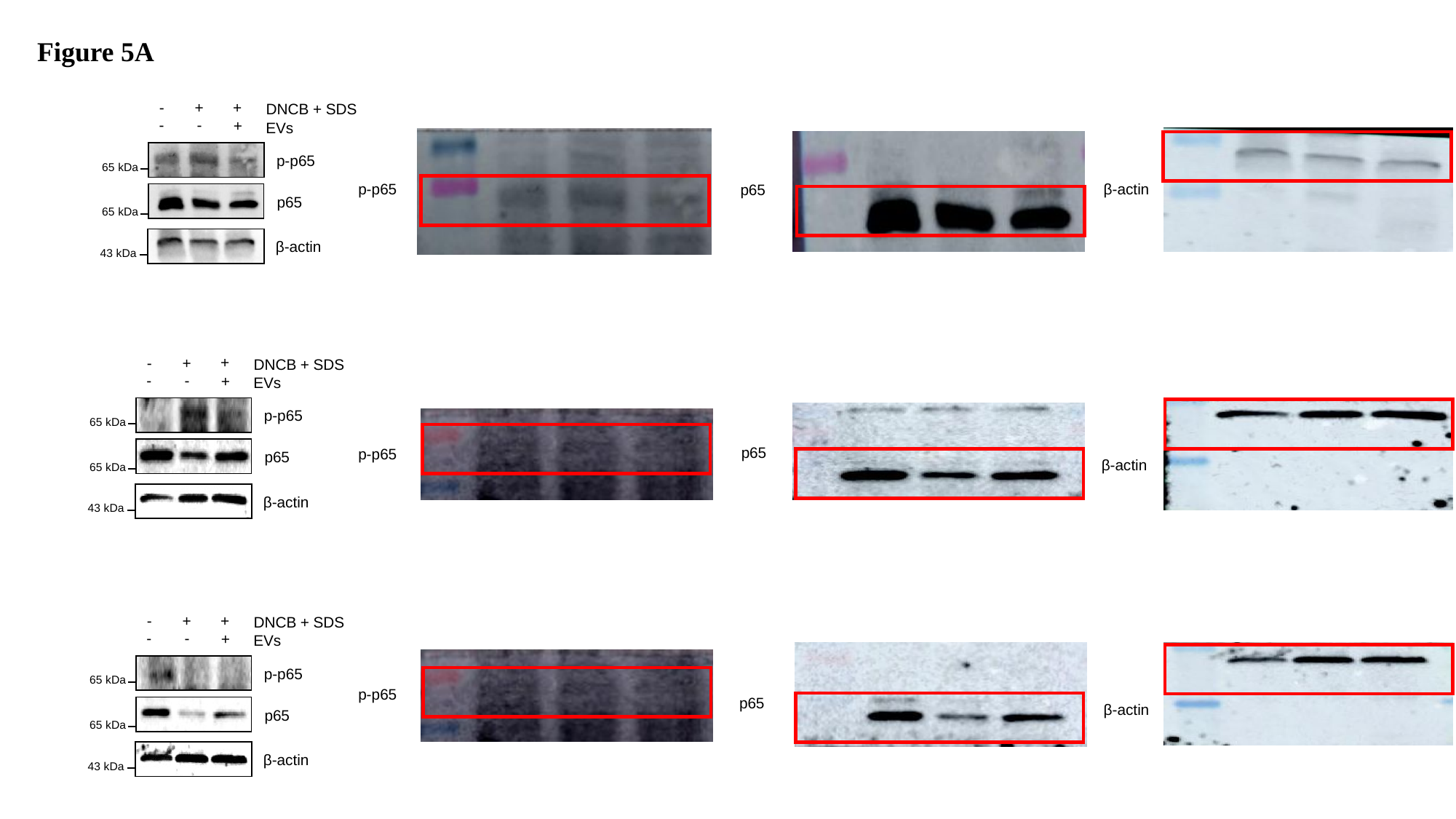

Figure 5A
+
-
+
DNCB + SDS
-
-
+
EVs
p-p65
65 kDa
p65
65 kDa
β-actin
43 kDa
β-actin
p-p65
p65
+
-
+
DNCB + SDS
-
-
+
EVs
p-p65
65 kDa
p65
65 kDa
β-actin
43 kDa
p65
p-p65
β-actin
+
-
+
DNCB + SDS
-
-
+
EVs
p-p65
65 kDa
p65
65 kDa
β-actin
43 kDa
p-p65
p65
β-actin

## Slide 9
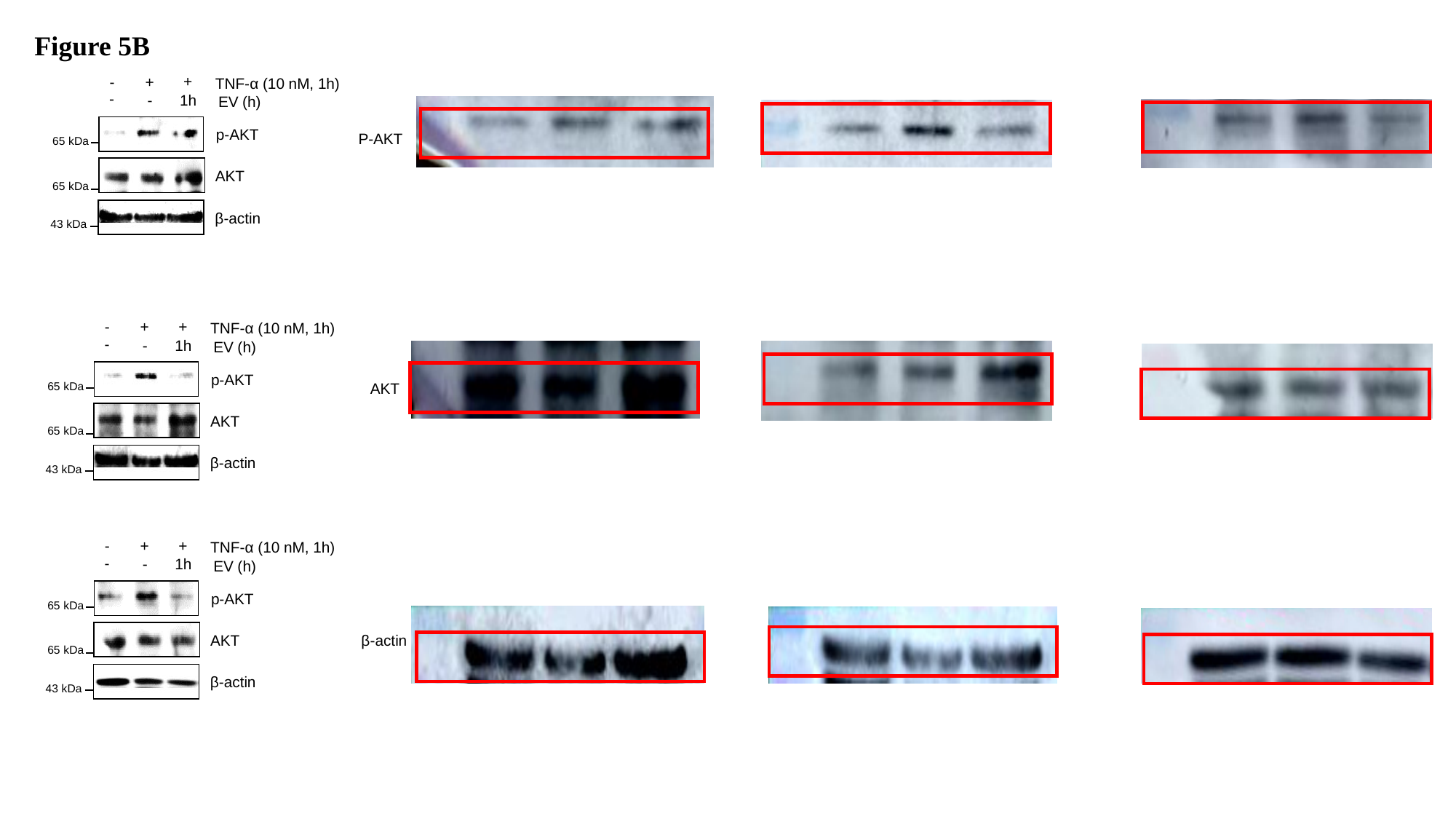

Figure 5B
+
-
+
TNF-α (10 nM, 1h)
-
1h
-
 EV (h)
p-AKT
65 kDa
AKT
65 kDa
β-actin
43 kDa
P-AKT
+
-
+
TNF-α (10 nM, 1h)
-
1h
-
 EV (h)
p-AKT
65 kDa
AKT
65 kDa
β-actin
43 kDa
AKT
+
-
+
TNF-α (10 nM, 1h)
-
1h
-
 EV (h)
p-AKT
65 kDa
AKT
65 kDa
β-actin
43 kDa
β-actin

## Slide 10
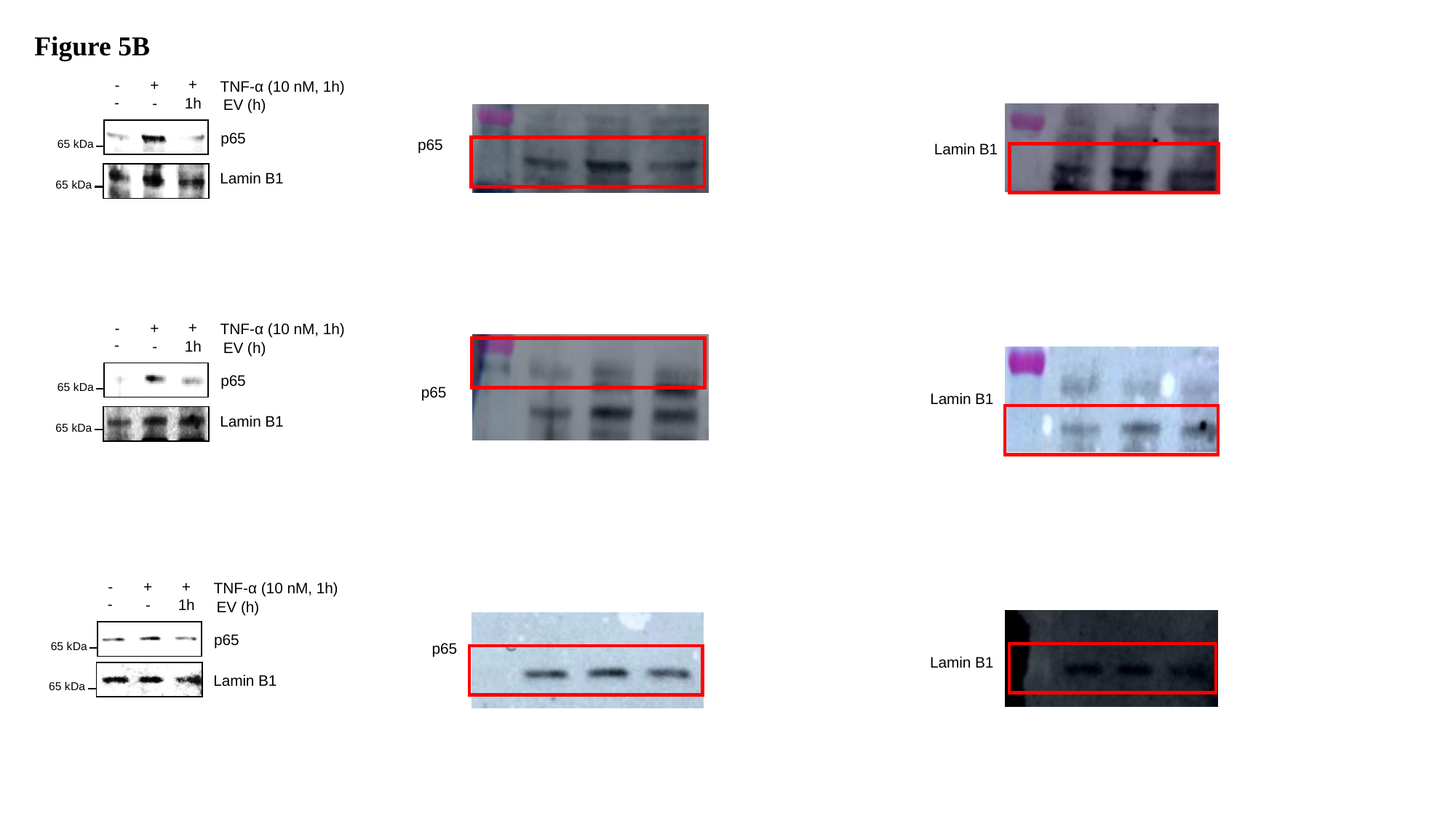

Figure 5B
+
-
+
TNF-α (10 nM, 1h)
-
1h
-
 EV (h)
p65
65 kDa
Lamin B1
65 kDa
p65
Lamin B1
+
-
+
TNF-α (10 nM, 1h)
-
1h
-
 EV (h)
p65
65 kDa
Lamin B1
65 kDa
p65
Lamin B1
+
-
+
TNF-α (10 nM, 1h)
-
1h
-
 EV (h)
p65
65 kDa
Lamin B1
65 kDa
p65
Lamin B1

## Slide 11
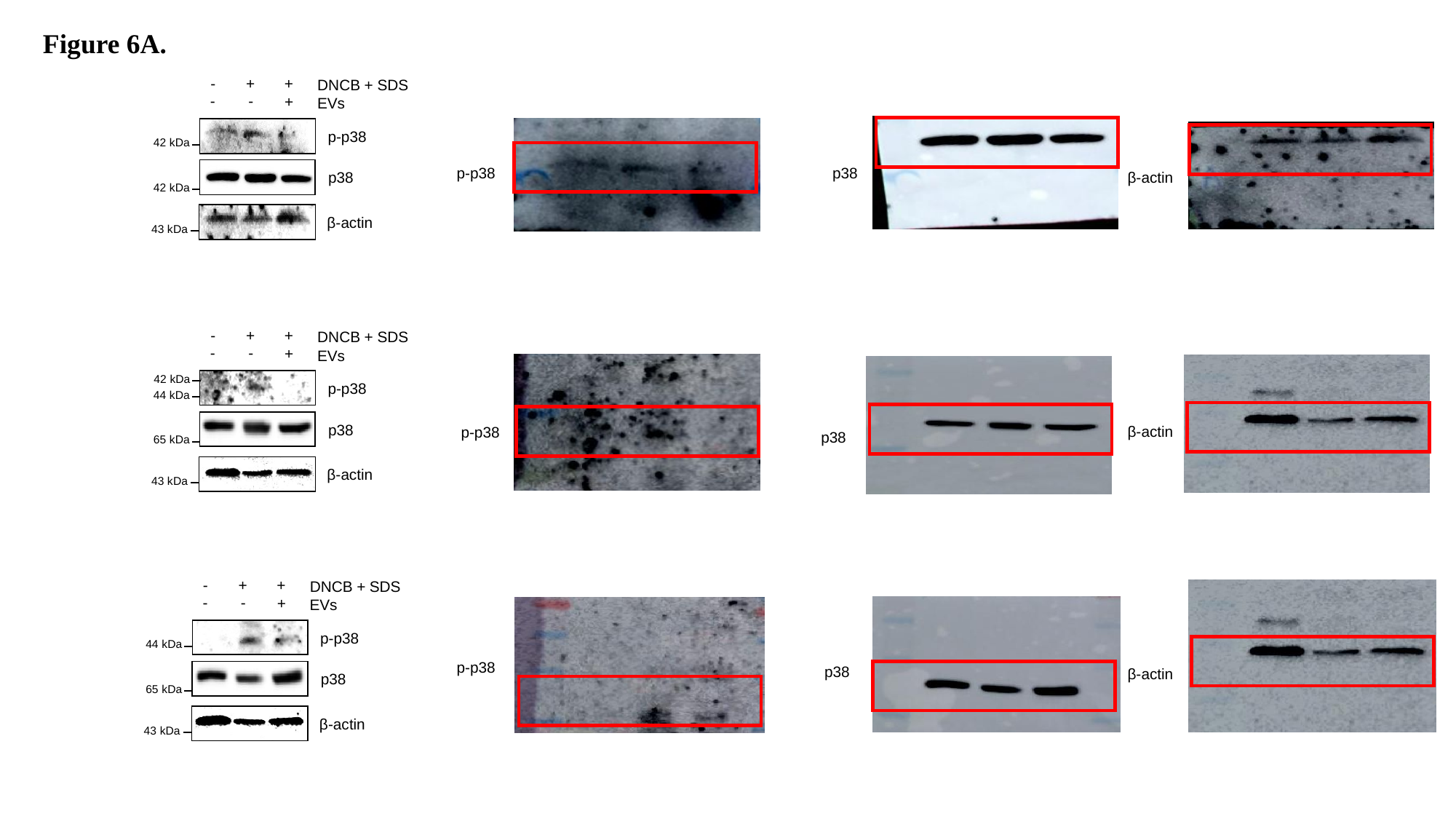

Figure 6A.
+
-
+
DNCB + SDS
-
-
+
EVs
p-p38
42 kDa
p38
42 kDa
β-actin
43 kDa
p38
p-p38
β-actin
+
-
+
DNCB + SDS
-
-
+
EVs
42 kDa
p-p38
44 kDa
p38
65 kDa
β-actin
43 kDa
β-actin
p-p38
p38
+
-
+
DNCB + SDS
-
-
+
EVs
p-p38
44 kDa
p38
65 kDa
β-actin
43 kDa
p-p38
p38
β-actin

## Slide 12
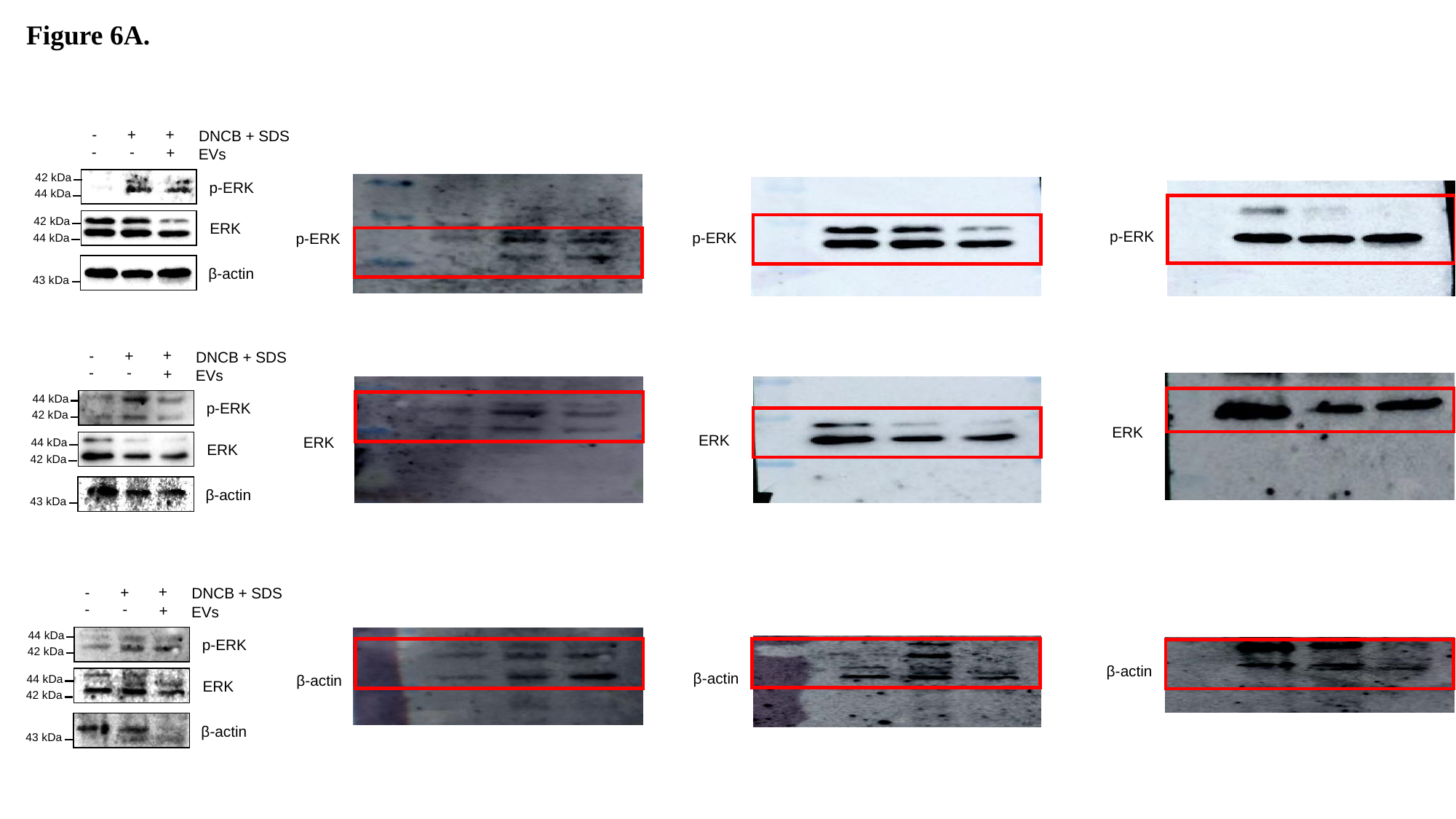

Figure 6A.
+
-
+
DNCB + SDS
-
-
+
EVs
42 kDa
p-ERK
44 kDa
42 kDa
ERK
44 kDa
β-actin
43 kDa
p-ERK
p-ERK
p-ERK
+
-
+
DNCB + SDS
-
-
+
EVs
44 kDa
p-ERK
42 kDa
44 kDa
ERK
42 kDa
β-actin
43 kDa
ERK
ERK
ERK
+
-
+
DNCB + SDS
-
-
+
EVs
44 kDa
p-ERK
42 kDa
44 kDa
ERK
42 kDa
β-actin
43 kDa
β-actin
β-actin
β-actin

## Slide 13
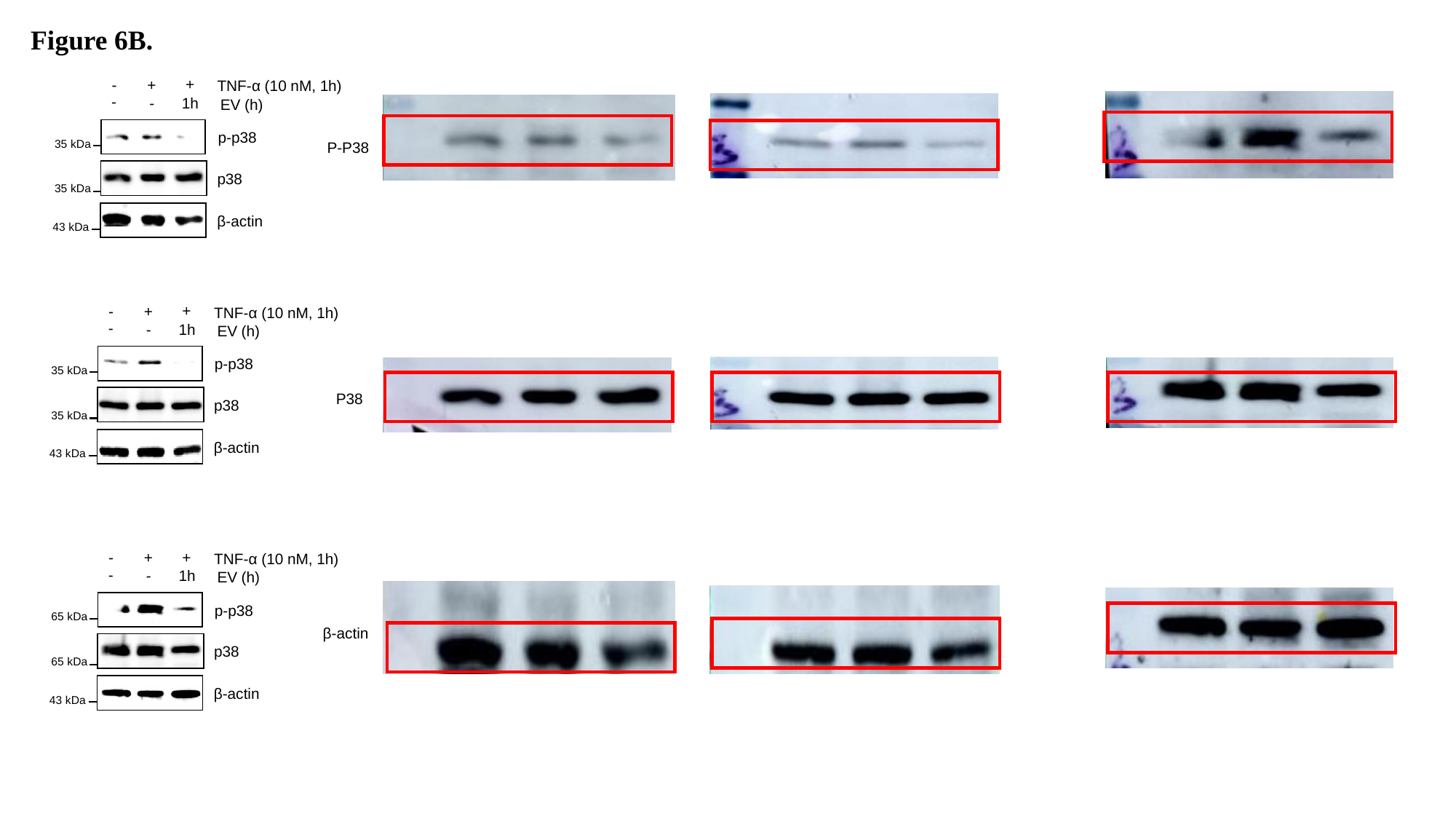

Figure 6B.
+
-
+
TNF-α (10 nM, 1h)
-
1h
-
 EV (h)
p-p38
35 kDa
P-P38
p38
35 kDa
β-actin
43 kDa
+
-
+
TNF-α (10 nM, 1h)
-
1h
-
 EV (h)
p-p38
35 kDa
p38
35 kDa
β-actin
43 kDa
P38
+
-
+
TNF-α (10 nM, 1h)
-
1h
-
 EV (h)
p-p38
65 kDa
p38
65 kDa
β-actin
43 kDa
β-actin

## Slide 14
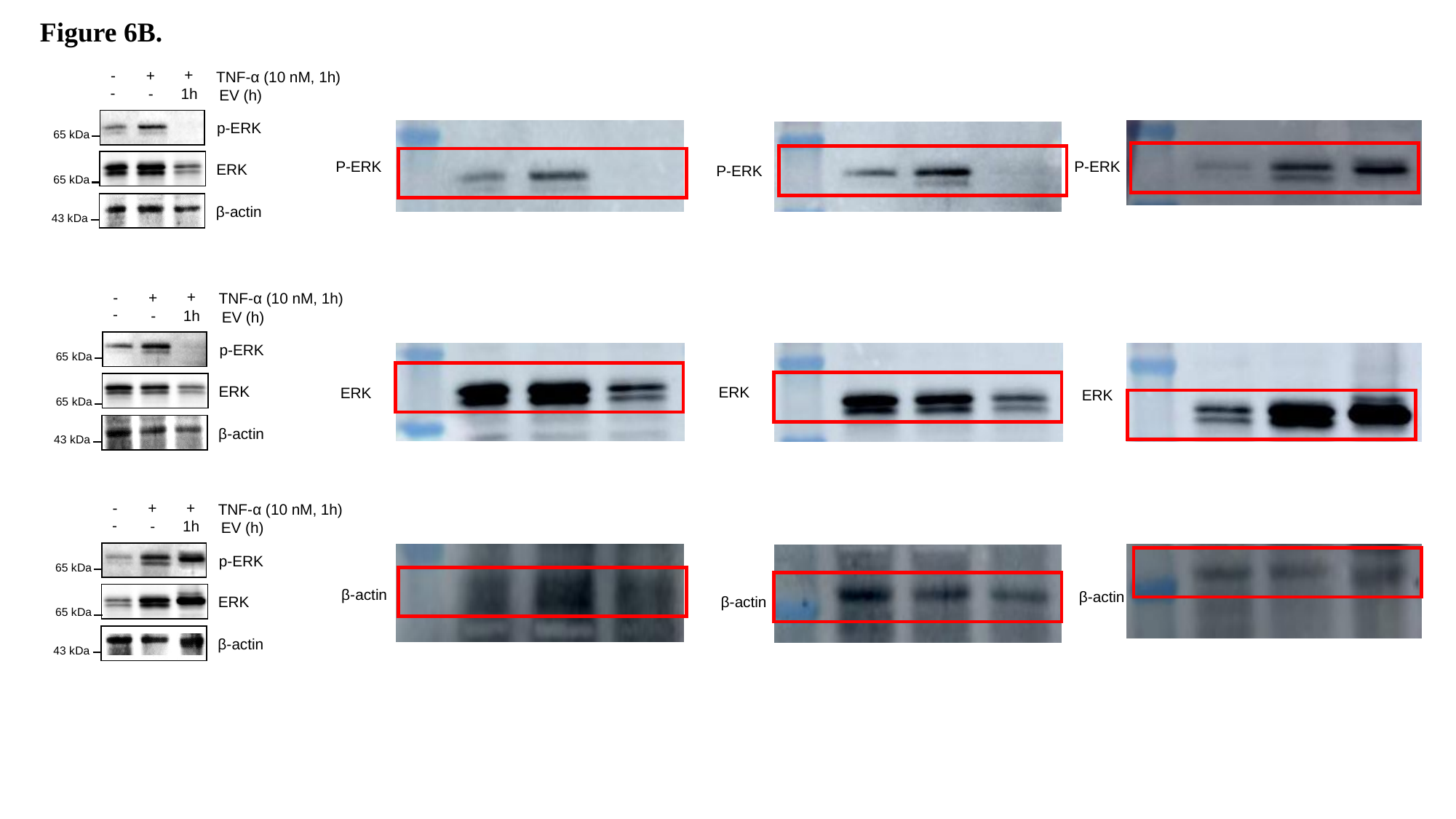

Figure 6B.
+
-
+
TNF-α (10 nM, 1h)
-
1h
-
 EV (h)
p-ERK
65 kDa
ERK
65 kDa
β-actin
43 kDa
P-ERK
P-ERK
P-ERK
+
-
+
TNF-α (10 nM, 1h)
-
1h
-
 EV (h)
p-ERK
65 kDa
ERK
65 kDa
β-actin
43 kDa
ERK
ERK
ERK
+
-
+
TNF-α (10 nM, 1h)
-
1h
-
 EV (h)
p-ERK
65 kDa
ERK
65 kDa
β-actin
43 kDa
β-actin
β-actin
β-actin

## Slide 15
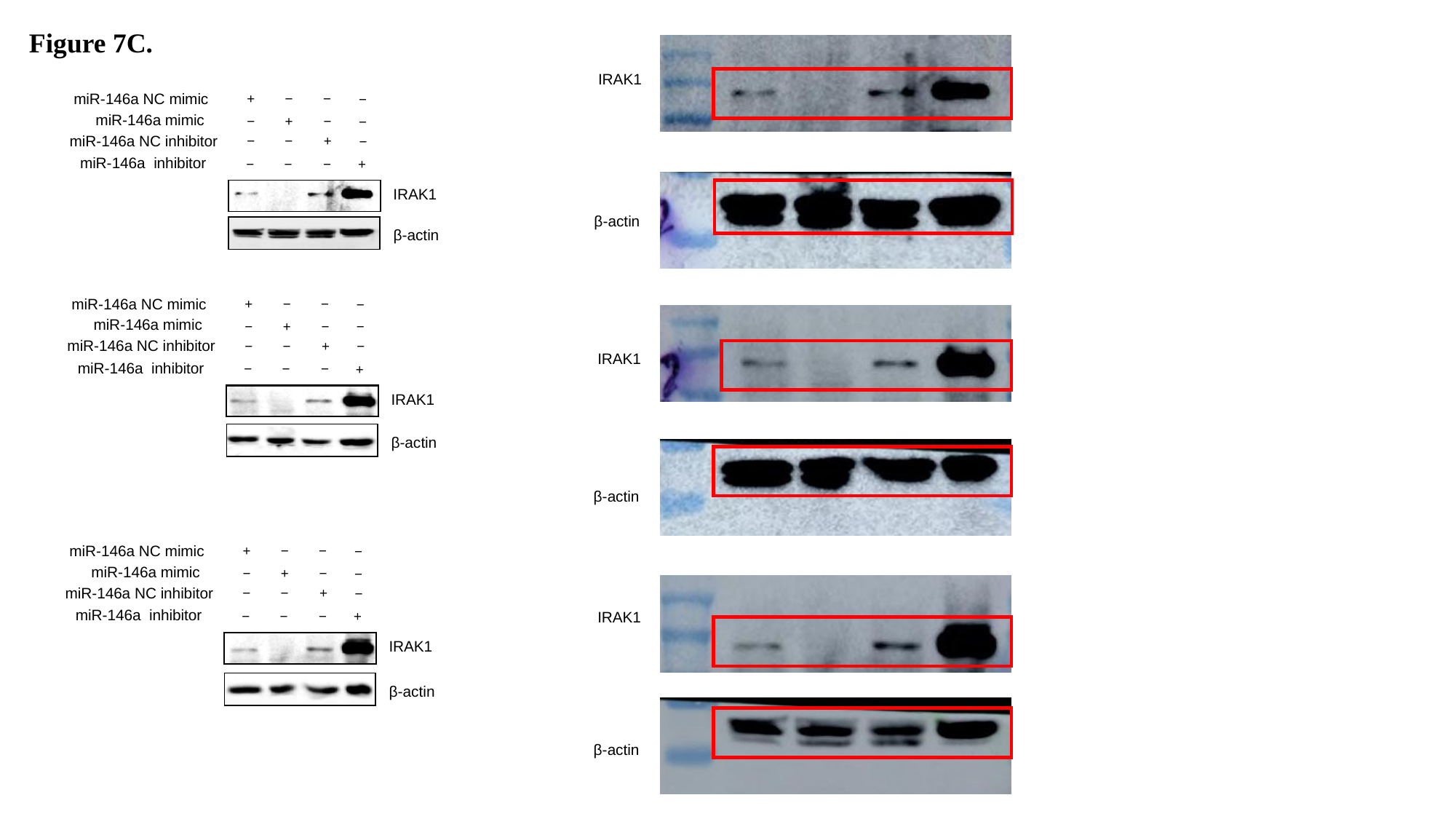

Figure 7C.
IRAK1
+
−
−
−
miR-146a NC mimic
−
+
−
miR-146a mimic
−
−
−
+
−
miR-146a NC inhibitor
−
−
−
+
miR-146a inhibitor
IRAK1
β-actin
β-actin
+
−
−
−
miR-146a NC mimic
−
+
−
miR-146a mimic
−
−
−
+
−
miR-146a NC inhibitor
−
−
−
+
miR-146a inhibitor
IRAK1
β-actin
IRAK1
β-actin
+
−
−
−
miR-146a NC mimic
−
+
−
miR-146a mimic
−
−
−
+
−
miR-146a NC inhibitor
−
−
−
+
miR-146a inhibitor
IRAK1
β-actin
IRAK1
β-actin

## Slide 16
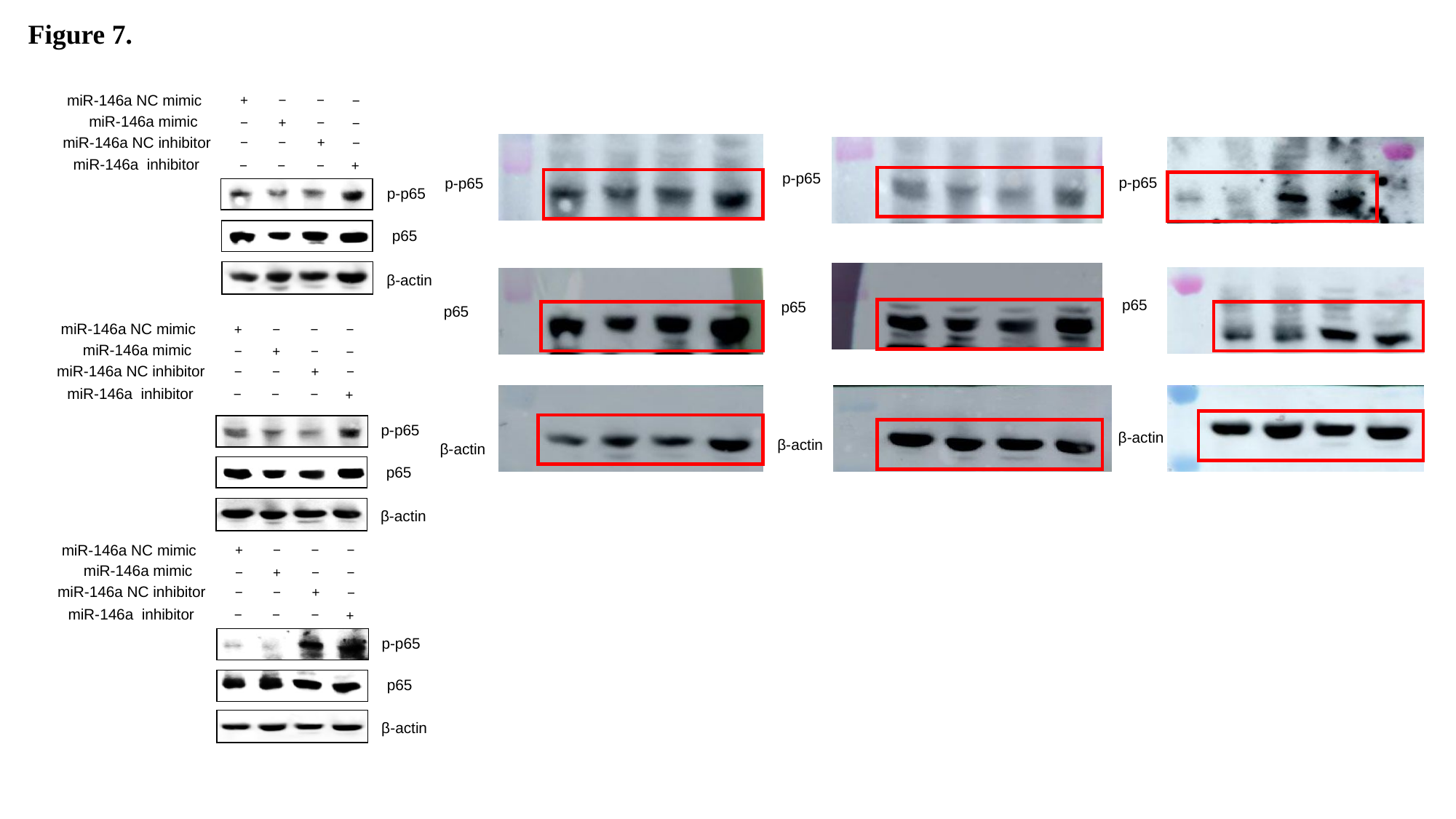

Figure 7.
+
−
−
−
miR-146a NC mimic
−
+
−
miR-146a mimic
−
−
−
+
−
miR-146a NC inhibitor
−
−
−
+
miR-146a inhibitor
p-p65
p65
β-actin
p-p65
p-p65
p-p65
p65
p65
p65
+
−
−
−
miR-146a NC mimic
−
+
−
miR-146a mimic
−
−
−
+
−
miR-146a NC inhibitor
−
−
−
+
miR-146a inhibitor
p-p65
p65
β-actin
β-actin
β-actin
β-actin
+
−
−
−
miR-146a NC mimic
−
+
−
miR-146a mimic
−
−
−
+
−
miR-146a NC inhibitor
−
−
−
+
miR-146a inhibitor
p-p65
p65
β-actin
